# B cell-extrinsic and intrinsic factors linked to early immune repletion after anti-CD20 therapy in patients with multiple sclerosis of African ancestry

**DOI:** 10.3389/fimmu.2025.1590165

**Published:** 2025-06-10

**Authors:** Gregg J. Silverman, Abhimanyu N. Amarnani, Arnaldo A. Arbini, Angie Kim, Hannah Kopinsky, David Fenyo, Ilya Kister

**Affiliations:** ^1^ Department of Medicine, Division of Rheumatology, NYU Grossman School of Medicine, New York, NY, United States; ^2^ Department of Pathology, NYU Grossman School of Medicine, New York, NY, United States; ^3^ Department of Neurology, NYU Grossman School of Medicine, New York, NY, United States; ^4^ Department of Biochemistry and Molecular Pharmacology, NYU Grossman School of Medicine, New York, NY, United States

**Keywords:** Ocrelizumab, multiple sclerosis, rituximab, CD20, African ancestry, B-cell repletion, genetics, B-cell activating factor

## Abstract

**Introduction:**

Recent investigations have identified patients of African ancestry (AA) with Multiple Sclerosis (MS), who display more rapid B-cell repopulation after standard semi-annual infusions with an anti-CD20 monoclonal antibody for B cell depletion. In this study, we explored the immunologic and genetic factors, with serum drug monitoring, that may contribute to a faster rate of B-cell repletion that follows during recovery from treatment with anti-CD20 antibody.

**Methods:**

In AA MS patients treated with an anti-CD20 antibody that had early repopulation of peripheral blood B cells, we assessed for extrinsic factors, including the presence of anti-drug antibodies against ocrelizumab, which may contribute to early repletion. We also documented the associated serum drug levels. In addition, we examined for inheritance of intrinsic gene polymorphisms associated with B cell survival and immune function.

**Results:**

Our findings identified a subset of AA patients with early B cell repletion after anti-CD20 treatment associated with anti-drug antibodies and an absence of detectable drug. Furthermore, a separate set of AA patients with the early B cell repletion phenotype without anti-drug antibodies had significant over-representation of genetic polymorphisms that map to genes for the B cell survival factor, BAFF, to antibody-dependent cytotoxicity, and to pathways involved in inflammation, leukocyte activation and B cell differentiation.

**Discussion:**

In AA patients with MS, after anti-CD20 antibody treatment we found an unexpected high occurrence of early B cell replenishment. This was associated with the presence of anti-drug antibodies and/or specific genetic polymorphisms. Larger studies are now needed to determine whether these factors may lead to impaired therapeutic benefits of B cell targeted therapy and clinical progression, and these findings may be useful to guide future optimized personalized therapeutic strategies.

## 1 Introduction

Multiple sclerosis (MS) is a chronic demyelinating disease that affects the central nervous system (CNS), which was traditionally viewed as largely being a ‘T-cell mediated’ disorder, partly because of the predominance of T cells in demyelinating plaques. However, it is now recognized that MS-related pathological processes involve interactions between several immune cell types, with increasing evidence that antibody-independent functions of B cells play key roles in pathogenesis (1–5). Suspicion of the potential roles of B-cells in MS was first suggested based on oligoclonal immunoglobulin bands in the CSF of MS patients (4, 5) and later confirmed by the clinical efficacy of B-cell-depleting therapies in suppressing relapses and new MRI lesions in MS (6, 7). These treatments involve regular antibody injections or infusions, resulting in B cell depletion in the bloodstream. Yet, between patients there is variability in the time to B cell repopulation of the circulation, and concerns regarding the level of tissue B cell depletion at the site of disease.

MS affects individuals from different races and ethnicities (8). Recent studies indicate that the incidence of MS in the US is highest among women of African ancestry (AA) (9), yet MS patients of African descent remain understudied (10). B-cell depletion as a therapeutic strategy in autoimmune diseases began with the introduction of rituximab, a monoclonal antibody targeting CD20 on B cells. Rituximab has been widely used and studied for its efficacy in conditions such as rheumatoid arthritis and MS, leading to the development of other anti-CD20 agents (11). In our large and diverse urban MS cohort, we found that African ancestry was often a predictor of faster B-cell repletion among anti-CD20 therapy-treated patients (12). To investigate the mechanisms responsible for early repletion, we have performed a range of cellular, serum antibody, immune factor assays, and genetic analyses in AA MS patients with and without early repletion. The clinical consequences of early B cell repletion were also considered.

## 2 Materials and methods

### 2.1 Clinical and demographic characteristics and selection criteria

This study was approved by NYU Langone IRB. All patients signed informed consent. Eligible patients self-identified as AA and were receiving their neurologic care at the NYU MS Care Center. All but two patients met the 2017 revised McDonald Criteria (13) for relapsing-remitting MS, and these patients met the criteria for Neuromyelitis optica (NMO). All participants were receiving infusions of anti-CD20 antibody therapy (either rituximab or ocrelizumab) at the time of enrollment. All participants were recruited between July 2022 and May 2023. Demographic, clinical characteristics, treatment history, as well as details on relapses and MRI activity while on anti-CD20 therapy were abstracted from the medical record by two neurologists (HK and IK). Patients in our study experienced no major adverse events, including no grade three and above infusion reactions, while receiving anti-CD20 therapy. Patients were excluded if they had received high-dose steroids, intravenous immunoglobulin (IVIG), or plasma exchange within three months before screening or had other concurrent immunosuppressive therapy. We also excluded patients with a body mass index (BMI) of >40. ‘Early repleters’ were identified based on prior flow cytometry results obtained as part of clinical care. We defined a patient as an ‘Early Repleter’ (ER) if the percentage of CD19+ B cells in blood was >0.5% within 6 months after anti-CD20 infusion or >1.5% within 7 months after anti- CD20 infusion. Where possible, early repleters were matched to ‘Normal Repleter’ (NR) – patients who did not meet the criteria for early repletion – based on age, sex, type of anti-CD20 therapy (ocrelizumab, OCR, or rituximab, RTX), duration of anti-CD20 therapy, and BMI.

### 2.2 Sample processing

Peripheral blood samples were collected from all participants during their bi-annual clinic visit that was scheduled before the anti-CD20 infusion. Sera were separated, and peripheral blood mononuclear cells (PBMCs) were isolated by density-gradient centrifugation. Isolated PBMCs were then aliquoted and cryostored in liquid nitrogen until further analysis.

### 2.3 Flow cytometric analyses

PBMC samples were thawed, stained, and studied on the same day. Cells were also stained with Live/Dead Fixable Blue Dead Cell Stain kit (ThermoFisher Scientific), and Fc receptors were blocked (Human TruStain FcX, BioLegend), as per manufacturer’s instructions. Surface staining was performed, as well as intracellular staining with FOXP3 Fix/Perm kit (ThermoFisher Scientific).

Cells were washed, fixed, and data were acquired on a 5-laser (355/405/488/561/637 nm) SONY ID7000 Spectral analyzer. 250,000 lymphocytes per sample were collected. Immune cell subsets assessed included naïve T cells (CD3+CD4+), regulatory T cells (CD3+CD25+CD127low), T peripheral helper cells (CD3+CD4+PD1+CXCR5-), classical monocytes (CD14+CD16-), non-classical monocytes (CD14-CD16+), and classical NK cells (CD56dim CD16+), as well as naïve B cells (CD19+CD27-IgD+), switched memory B cells (CD19+CD27+IgD-), unswitched memory B cells (CD19+CD27+IgD+), double negative (CD19+CD27-IgD-) B cells. Populations were quantitated based on established phenotyping criteria. Data acquisition was performed using the FACSDiva (Becton-Dickinson), with analysis using FlowJo (v10.10) software. The gating strategy is shown in **Supplementary Figure S1**. All surface markers, fluorophores, and antibody clones are listed in **Supplementary Table S1**. All data were reviewed by a board-certified hematopathologist (AAA).

### 2.4 Serologic immunoassays testing

Serum B-cell activating factor (BAFF) (R&D Labs, PA), soluble CD40 ligand (sCD40L) (R&D Labs), and B-cell maturation antigen (BCMA) (R&D Labs) levels were measured by ELISA, according to the manufacturer’s instructions. In the OCR-treated patients with rapid repletion of B cell levels after infusion, serum OCR, and anti-OCR levels were evaluated. In RTX-treated ER patients, anti-RTX antibody levels (Sanguin, NL) were tested. Serum immunoglobulin quantitations were performed by ELISA with comparison to standard curves (Jackson Immunoresearch).

### 2.5 Statistical analyses of genetic SNP

Genetic variation was analyzed using the Infinium Immunoarray-24 v2 BeadChip (Illumina), a genotyping array that detects 247,814 single nucleotide polymorphisms (SNPs) related to immune system processes. To mitigate the high risk of false-positive findings inherent in assessing this large number of SNPs across our limited sample size, we implemented a multifaceted approach. In our analyses, we only considered SNPs for further analysis when they were present in at least two ER patients. Subsequently, we used g:SNPense (g:Profiler) (14) to map SNPs to gene names and assess these variants’ potential effects on gene function, as defined by Sequence Ontology. To further identify potentially relevant SNPs, we employed Fisher’s exact tests for association analysis, and we calculated the odds ratio (OR) confidence intervals for effect size estimation. We also compared the mean allele frequencies between the ER and NR groups. This multi-step approach, outlined in **Supplementary Figure S2**, aimed to identify biologically relevant SNPs while minimizing the risk of spurious associations.

Comparative analyses of demographic variables and clinical characteristics between ER and NR patients were performed using chi-square tests for categorical variables and non-parametric t-tests (Mann-Whitney U testing) for continuous variables. Fisher’s exact test was applied to assess the association between SNPs and repletion status (ER vs. NR) in a 2 x 3 table of germline heterozygous and homozygous alleles. Odds ratios (ORs) were calculated for homozygous vs. germline and heterozygous vs. germline SNPs. SNPs of particular interest were those that had a differential overrepresentation in ER vs. NR, as defined by Fisher’s exact test p-value < 0.05 and a lower confidence interval >1 for the odds ratio. All statistical analyses and visualizations were conducted using GraphPad Prism (Version 10.2.3) or R software (Version R 4.3.3).

### 2.6 Pathway analyses

To identify genes related to immune response and B cell differentiation that were significantly enriched in the ER group, pathway analysis were conducted using g:Profiler (14). Hypergeometric calculations were used to determine the significance of gene set overlaps. A rank-ordered list of genes, which were identified based on the SNPs overrepresented in the ER group, was entered into the g:Profiler algorithm. In ER patients, top priority was assigned to genes associated with more than one SNP overrepresented, with a lower ranking based on the highest minor allele frequency (MAF). This approach enabled the systematic identification of gene SNP-associated pathways involved in immune system responses. To consider the relevance of a gene SNP for B cell development and differentiation, we used a reference list of 8803 genes that was compiled from reports that defined genes differentially expressed during critical stages of B cell development (15–18).

## 3 Results

### 3.1 Patient characteristics and disease course in normal repleters and early repleters

We enrolled 20 AA MS patients who met our criteria for ER. All were diagnosed with MS, and 80% were female, with a mean age of 38.7 years [SD=12.9]. The mean duration of anti-CD20 therapy was 4.9 years [range: 2–8 years]. We enrolled 23 AA patients who were NR, of whom 21 had MS and 2 had NMO, 87% were female, the mean age was 42.3 + 9.9 years, and the mean duration on anti-CD20 therapy was 4.9 years [range: 1.3–14 years]. There were no differences in the demographic and clinical characteristics of the two groups (**Tables 1**, **2**), and no major adverse events were recorded.

**Table 1:** Comparative analysis of demographics, lab values, and B cell repletion of early repleters and normal repleters following B cell depletion therapy.

Characteristic	All Analyzed Patients *N*=43	ER *n* = 20	NR *n* = 23	*p-*Value^a^
Age, years				0.44
Mean (SD)	40.6 (11.4)	38.7 (12.9)	42.3 (9.9)	
Median (range)	42 (16.0-57.0)	40.5 (16.0-56.0)	43.0 (24.0-57.0)	
Female, *n* (%)	36 (83.7)	16 (80.0)	20 (87.0)	0.44
Body mass index, mean (SD), kg/m²	29.6 (4.8)	29.7 (4.0)	29.4 (5.4)	0.73
Diagnosis, *n* (%)				0.12
Multiple sclerosis	41 (95.3)	20 (100.0)	21 (91.3)	
Neuromyelitis optica	2 (4.7)	0 (0)	2 (8.7)	
Disease duration, mean (SD), years	13.2 (8.6)	12.2 (8.9)	14.0 (8.5)	0.35
Ambulation status, *n* (%)				0.13
Fully ambulatory	30 (69.8)	17 (85.0)	13 (56.5)	
Impaired but no assistance	1 (2.3)	0 (0)	1 (4.3)	
Ambulatory with cane or walker	9 (20.9)	3 (15.0)	6 (26.1)	
Non-ambulatory	3 (7.0)	0 (0)	3 (13.0)	
Smoking status, *n* (%)				0.26
Current	2 (4.7)	2 (10.0)	0 (0)	
Former	8 (18.6)	4 (20.0)	4 (17.4)	
Never	33 (76.7)	14 (70.0)	19 (82.6)	
B-cell depletion therapy, *n* (%)				0.56
Rituximab	11 (25.6)	4 (20.0)	7 (30.4)	
Ocrelizumab	32 (74.4)	16 (80.0)	16 (69.6)	
Length of treatment on B-cell depleting therapy, mean (SD), years	4.9 (2.2)	4.9 (1.9)	5.0 (2.4)	0.81
History of prior disease modifying therapy, *n* (%)	34 (79.1)	17 (85.0)	17 (73.9)	0.47
Laboratory test results				
Time since last infusion, mean (SD), months	5.1 (1.8)	5 (1.1)	5.1 (2.3)	0.75
White blood cell count, mean (SD), 10³/uL	5.5 (2.0)	5.3 (2.1)	5.7 (2.0)	0.45
Percentage of CD19 positive cells, mean (SD)	2.2 (4.0)	4.4 (5.0)	0.2 (0.6)	**<0.0001**
Immunoglobulin A (IgA), mean (SD), mg/dL	225.9 (114.0)	266.6 (124.9)	188.9 (90.9)	**0.03**
Immunoglobulin G (IgG), mean (SD), mg/dL	1128.0 (388.3)	1233.0 (461.8)	1033.0 (285.2)	0.16
Immunoglobulin M (IgM), mean (SD), mg/dL	59.4 (41.6)	59.0 (42.8)	60.2 (41.5)	0.62

p-values <0.05 are shown in bold.

ER, early repleter; NR, normal repleter.^a^p-values represent chi-square tests for categorical variables and non-parametric t-tests (Mann-Whitney U testing) for continuous variables.

**Table 2:** Ten ER and six NR were among the participants who were tested for anti-ocrelizumab anti-drug antibodies (ADA), no NR participant has ADA, while two ER patients with ADA were identified.

Anti-OCR Antibody (AU/mL)	OCR Level (ug/mL)	% B Cell Repletion	Total IgG level (mg/dL)	Time Since Infusion (Months)	Additional Information
576	<0.0025	17%	3279	3-4	- Previous treatments: Fingolimod - Co-morbidities: SLE and Sjogren’s syndrome
		13%	2952	4-5	
		14%	2917	6-7	
101	<0.0025	4%	1728	4-5	- Previous treatments: Interferon beta-1a - Co-morbidities: Hyperlipidemia
		5.5%	1490	7-8	
		5.5%	1615	8-9	

The levels of these antibodies and the available post-infusion level of circulating CD19+ cells are provided for each patient. Both patients were female, and age 30–50 years old. OCR, ocrelizumab.

These two patients with detectable levels of anti-ocrelizumab antibodies (>87.5 AU/mL) are highlighted in the table.

Neurologist-diagnosed relapses while on anti-CD20 therapy occurred in 4/20 ER patients (20%), and the mean annual relapse rate (ARR) in the ER was 4.1%. Relapses while on anti-CD20 therapy were documented in 5 NR/23 patients (22%), with one patient having 2 relapses; in NR, the mean annual relapse rate was 5.3%, which is similar to the relapse rate of the ER patients (p=0.849). In ER patients, a total of 88 MRIs were obtained while on anti-CD20 therapy, and in NR patients - 91 MRIs. New MRI lesions were observed in 4/20 of the ER patients (20%; mean annual lesion formation rate of 4.1%) and in 3/23 of the NR patients (13%; mean annual lesion formation rate of 2.7%). There was no difference between new lesion rates in the two groups (p=0.796). Clinical and MRI activity for each of the participants throughout anti-CD20 therapy is shown in **Supplementary Figure S3**.

**Figure 1:**
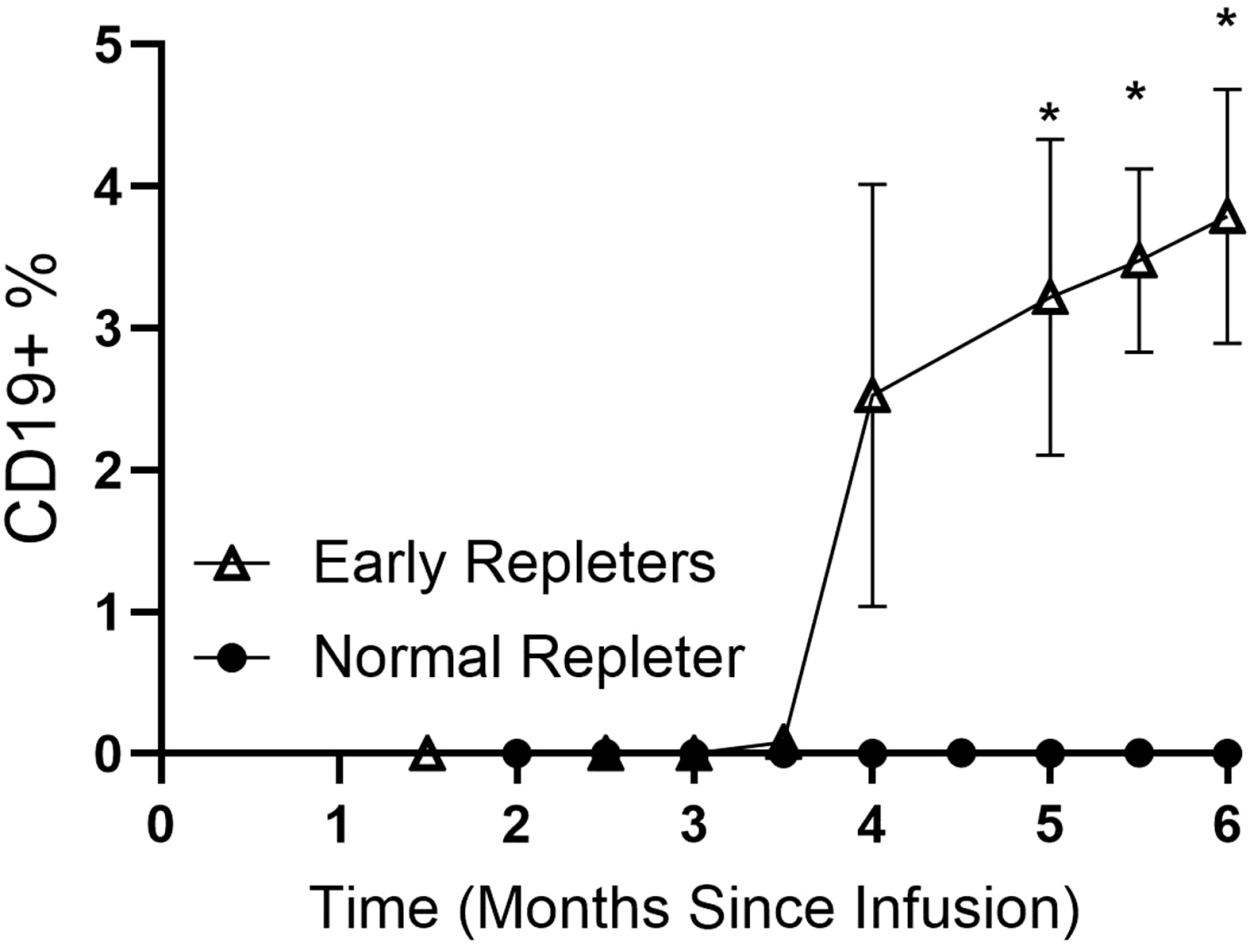
B cell repopulation post-CD20 depletion in early repleters (ER) and normal repleters (NR). Data for 23 NR and 20 ER MS patients are shown, with a total of 133 longitudinal samples collected over 1.5 to 6 months post anti-CD20 infusion. Values for CD19+ (B lineage) cells are percentages of total circulating lymphocytes. Data are presented as mean ± standard error of the mean. *indicates the specific time points attaining p<0.01 (Mann-Whitney U test).

ER and NR patients had similar intervals from the anti-CD20 infusion to blood sample collection (p=0.75) (**Table 1**). As expected, ER patients had significantly higher levels of CD19+ B cells compared to the NR (p <0.0001, **Table 1**). All ER patients had detectable levels of B-cells within 6 months post-infusion, while none of the NR patients had detectable circulating B cells within 6 months of their last anti-CD20 infusion (**Figure 1**). Serum IgA levels were significantly higher in the ER group compared to the NR group (p = 0.03), while serum IgG and IgM levels were not different (p = 0.35, p = 0.61, respectively) (**Table 1**, **Supplementary Figure S4**).

### 3.2 Anti-CD20 concentrations and anti-drug levels in ER and NR patients

To ascertain whether differences in rates of B-cell repletion in the two groups may be due to differences in drug bioavailability, we assessed OCR concentration levels in a subset of 17 OCR-treated patients (6 NR and 10 ER). There were no significant differences in the number of days between the blood collection and the last OCR infusion in the NR- and ER-tested patients (NS, p=0.62) and no differences in OCR concentrations in NR- and ER-tested patients (**Supplementary Figure S5A, B**).

We also assessed for the presence of anti-drug antibodies (ADA) (i.e., anti-OCR antibodies) in this same 17 patients and found detectable ADA levels in two OCR-treated ER patients. The two patients with anti-OCR ADA had no detectable OCR drug levels (<0.0025 ug/mL). B-cell repletion histories for the two patients with anti-OCR ADA are shown in **Table 2**.

Among the OCR-treated patients without ADA, there was no correlation between blood CD19+ B cell level and OCR (**Supplementary Figure S5C**). Sera from three out of 4 of the RTX-treated ER patients were available for anti-RTX antibody testing, and none had ADA.

**Figure 2:**
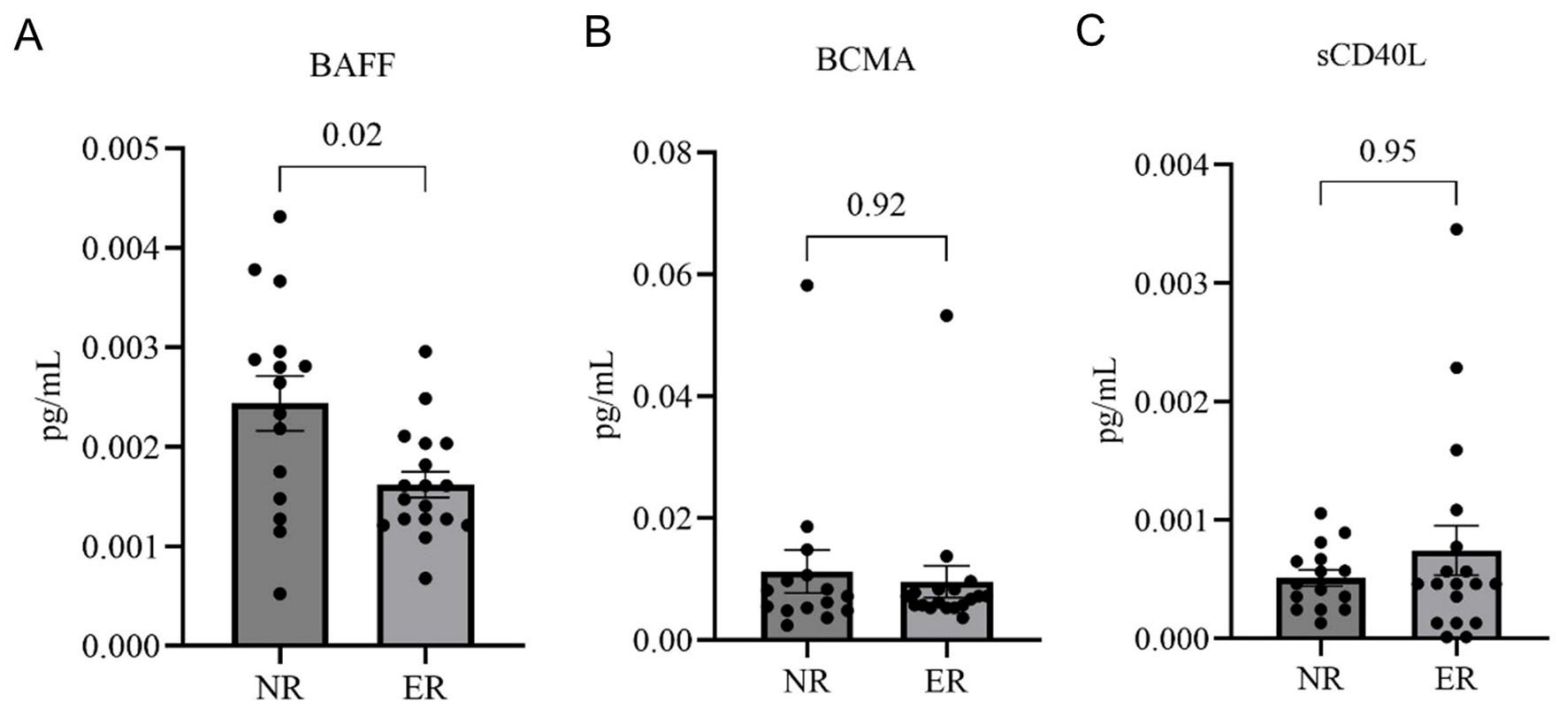
Serum levels of BAFF, sCD40L, and BCMA in normal repleters (NR, n=15) vs. early repleters (ER, n=18). **(A)** Serum levels of B-cell activating factor (BAFF) are significantly higher in the NR group (p=0.02). **(B)** Soluble CD40 ligand (sCD40L) levels were not different, and **(C)** B-cell maturation antigen (BCMA) were also not different between ER and NR groups. All serum samples were drawn between 3 and 6 months post-ocrelizumab treatment. Quantification was performed using commercial ELISA. Statistical comparisons were made using the Mann-Whitney U test. Data represent mean ± standard error of the mean.

Levels of B-cell survival factors, but not B cell activation factors, differ between NR and ER patients Soluble factors can have dramatic systemic effects on B cell survival, differentiation, and activation, and we, therefore, examined serum levels of the TNF family member co-stimulation factor, sCD40 ligand (sCD40L), as well as the soluble form of the B cell maturation antigen (BCMA) that is shed by plasma cells. We found that these levels were similar in the ER and NR groups (**Figure 2**).

Based in part on a report of an MS cohort with overrepresentation of specific BAFF-related polymorphism that could lead to B-cell dysregulation (19), we next examined levels of the protein, B cell activation factor (BAFF), also termed B lymphocyte stimulator, that affects the survival of cells of the B lineage. We found that serum BAFF levels were significantly higher in NR than in the ER patients at 3–6 months post-infusion (p = 0.02) (**Figure 2**). This pattern was expected, as reciprocal systemic BAFF elevations are known to be concurrent with the overall depletion of B-lineage cells (20). The lower BAFF levels in ER patients are likely to reflect the greater consumption of BAFF because of more rapid repopulation and differentiation of the peripheral B-cell pool and, therefore, serve as independent confirmation of early repleter status. No significant correlation of BAFF, BCMA, or sCD40L with the time interval since infusion was observed (**Supplementary Figure S6**).

### 3.3 Representation of B cell subsets in ER and NR groups

Complete blood cell counts were assessed at a mean of 4.9 (± 1.9) months after the last anti- CD20 infusion. All samples contained more than 1 x 10^9 total lymphocytes/mL. Between the ER and NR groups, there were no significant differences in the representation in the peripheral blood of CD4+ T cells, CD8+ T cells, regulatory T cells (CD4+CD25+), or T peripheral helper cells (TPH) (CD4+PD1+CXCR5-) (**Supplementary Figure S6**).

We also examined the representation of B lymphocyte subsets, defined as CD19-bearing, in peripheral blood samples collected after anti-CD20 infusions. Notably, whereas B cell repletion was more rapid in the bloodstream of the ER group (**Figure 1**), the representation of different B cell phenotypic subsets was comparable, with no significant differences in the percentages of mature (CD27-sIgD+), switched memory (CD27+IgD-), unswitched memory (CD27+IgD+), or Double Negative (CD27- sIgD-) B cells in the ER compared to the NR group, relative to the total number of B cells, which suggested these two groups displayed similar repopulation dynamics. In both ER and NR, transitional and immature B cells, which are the subsets that newly arise to enter the circulation, were the dominant subsets (>95% in both NRs and ERs) (**Supplementary Figure S7**).

### 3.4 Genetic variation and SNP analysis

We next investigated for differences in the inherited genetic variants with possible involvement in early B cell repopulation. For the genetic analyses, the two patients with raised anti-OCR antibodies were omitted, as immune-mediated B cell clearance mechanisms were sufficient to account for earlier B-cell repletion in these two subjects. We therefore explored the genetic factors that potentially contribute to early B cell repletion in AA MS, using the Immunoarray Beadchip (Illumina) and performing case-control association analysis. Out of the 247,814 SNPs tested, 189,777 SNPs were found in at least two of the ER patients in our cohort. Of these, there was a significant overrepresentation of 6,471 SNPs in the ER. Furthermore, of these, 3,724 had a higher mean allele frequency in ER (Fisher’s exact test, p < 0.05). We further determined the representation of homozygous and heterozygous SNPs in ER versus NR. This analysis identified 1,887 SNPs of interest (1,520 heterozygous vs. germline; 281 homozygous vs. germline; 84 both homozygous and heterozygous vs. germline) with the lower limit of the confidence interval for the odds ratios ≥1. Additionally, among the significantly overrepresented SNPs (Fisher’s exact test, p < 0.005) with a positive mean allele frequency in ERs compared to NRs, we identified 317 SNPs of interest that had a mean allele frequency for NRs but an allele frequency of at least 2in ERs (**Supplementary Table S2**).

**Figure 3:**
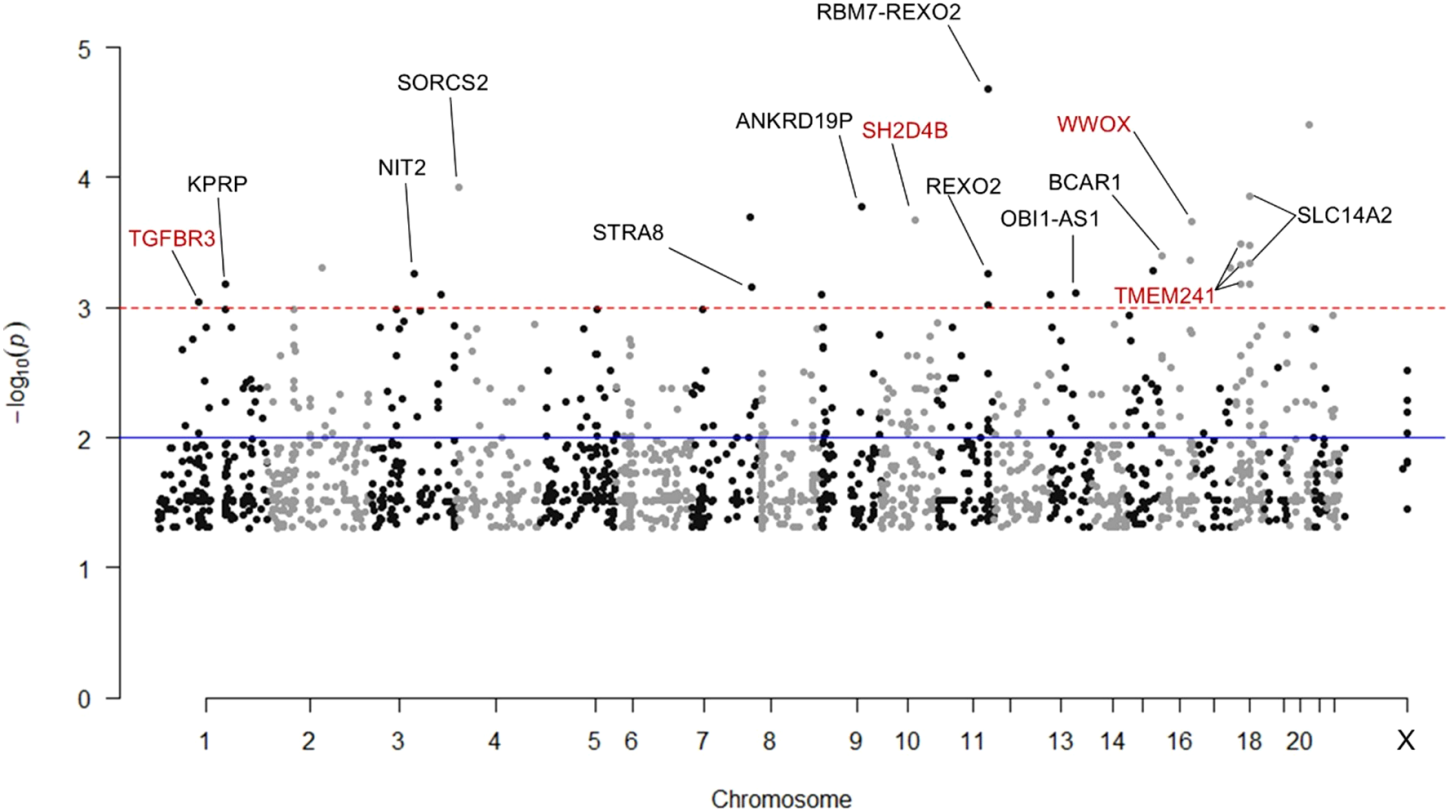
Manhattan plot of 2,202 SNPs significantly overrepresented in early repleters (ER) vs. normal repleters (NR). The plot displays SNPs identified as significantly overrepresented among ER participants by Fisher’s exact test (p-value < 0.05) and meeting at least one of three additional significance criteria: 1) Lower limit confidence interval > 1 for the odds ratio of homozygous to germline SNPs, 2) Lower limit confidence interval > 1 for the odds ratio of heterozygous to germline SNPs, 3) Mean allele frequency of for NRs with at least 2 SNP alleles identified in ERs. SNPs with a negative log10(p-value) > 3 that are mapped to genes by g:SNPense are annotated. Gene names in red indicate SNPs of interest related to immune system processes, particularly B cell activation and development.

**Figure 4:**
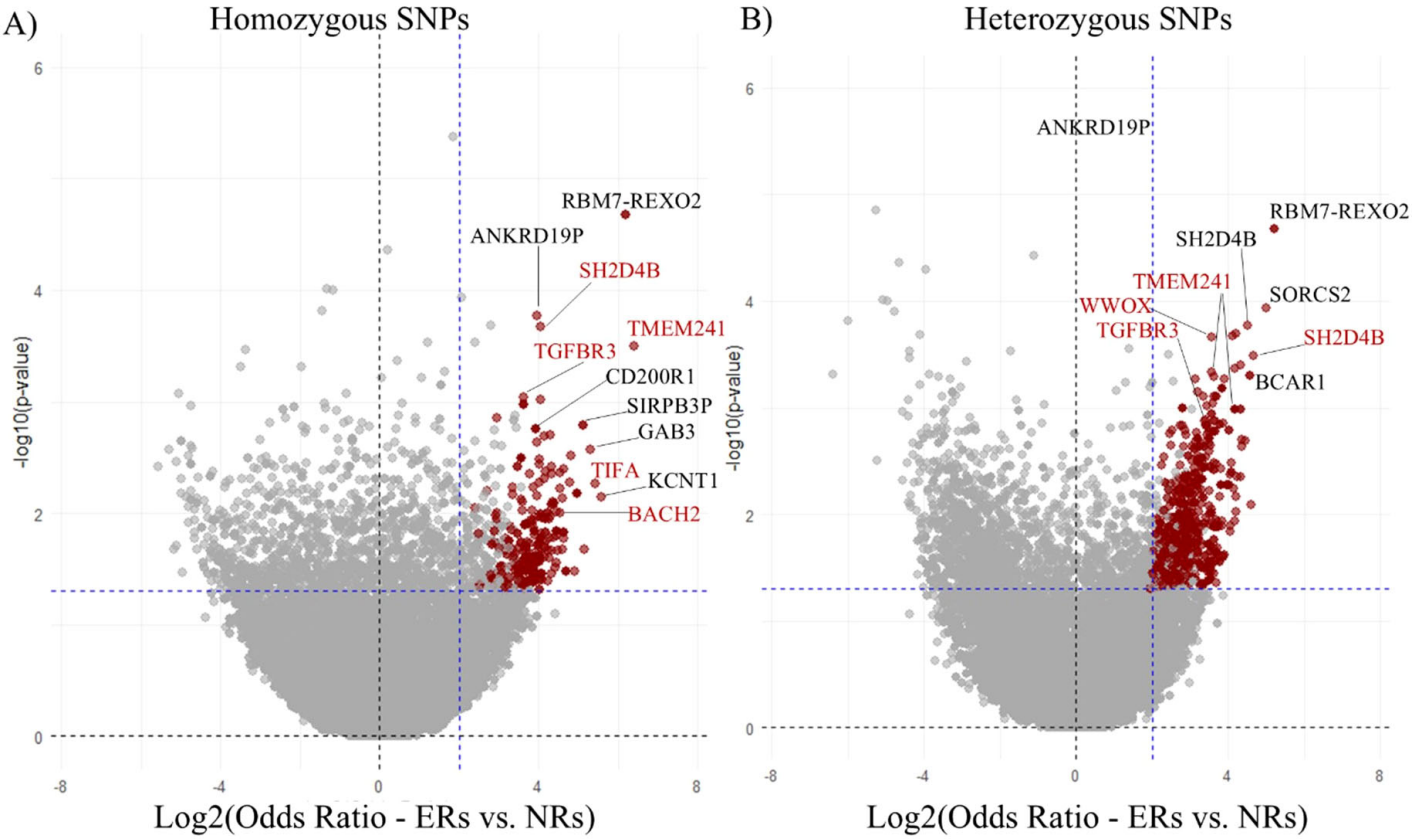
Odds ratios of SNPs in early repleters (ER) vs. normal repleters (NR). This figure presents the odds ratios of homozygous **(A)** and heterozygous **(B)** SNPs when comparing early repleters (ER) to normal repleters (NR). SNPs highlighted in red indicate those with a Fisher’s exact test p< 0.05, and an odds ratio lower limit >1 (365 for homozygous and 1,604 for heterozygous). The most overrepresented SNPs of interest are annotated with the names of their corresponding mapped genes (g:SNPense).

**Figure 5:**
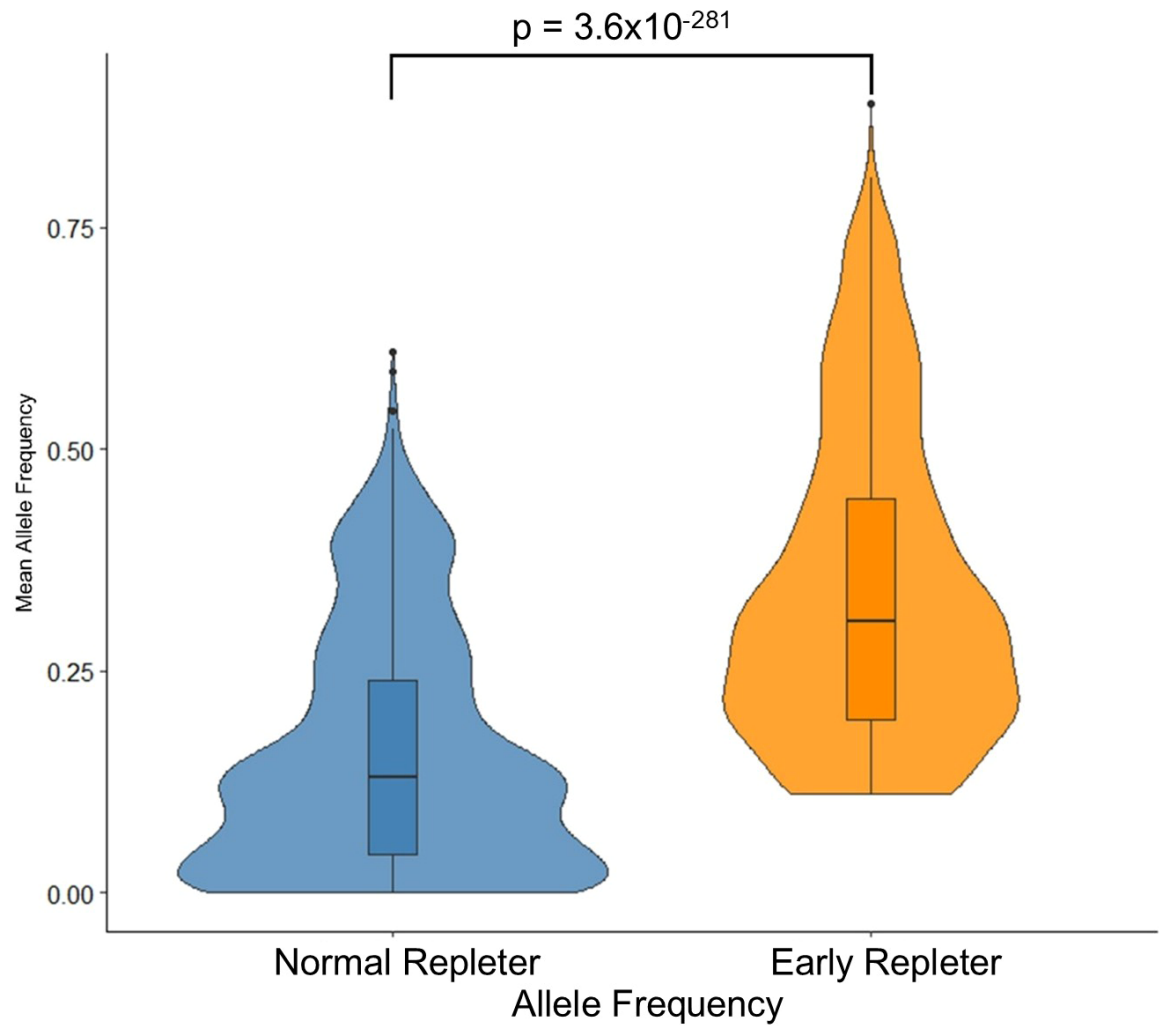
Distribution of allele frequencies in early repleter (ER) and normal repleter (NR) groups. The figure depicts the distribution of allele frequencies for 2,202 SNPs that were significantly overrepresented in the ER group. Violin plots show the density of allele frequencies, while the box plots indicate the median, quartiles, and potential outliers. Box and whisker plots represent the interquartile range (IQR), with the whiskers extending to 1.5 times the IQR from the first and third quartiles. Outliers are represented as individual points beyond the whiskers.

The distribution of SNPs across the genome is shown in **Figure 3**. Notably, four of the 2,202 SNPs overrepresented in ER, with a p-value <0.001, were associated with genes TGFBR3, SH2D4B, TMEM241, and WWOX, which are known to have roles in immune system function, including B cell development. A volcano plot illustrating the odds ratios for homozygous versus germline and heterozygous versus germline SNPs in ER versus NR is shown (**Figure 4**). Of SNPs of interest, the mean allele frequency was significantly higher in ERs than in NRs (**Figure 5**). Notably, these SNPs also had a higher MAF in ERs compared to reference control subjects of African descent described by Illumina (**Supplementary Figures S8A, B**).

### 3.5 Pathway analysis for SNPs with known gene variant effects

**Figure 6:**
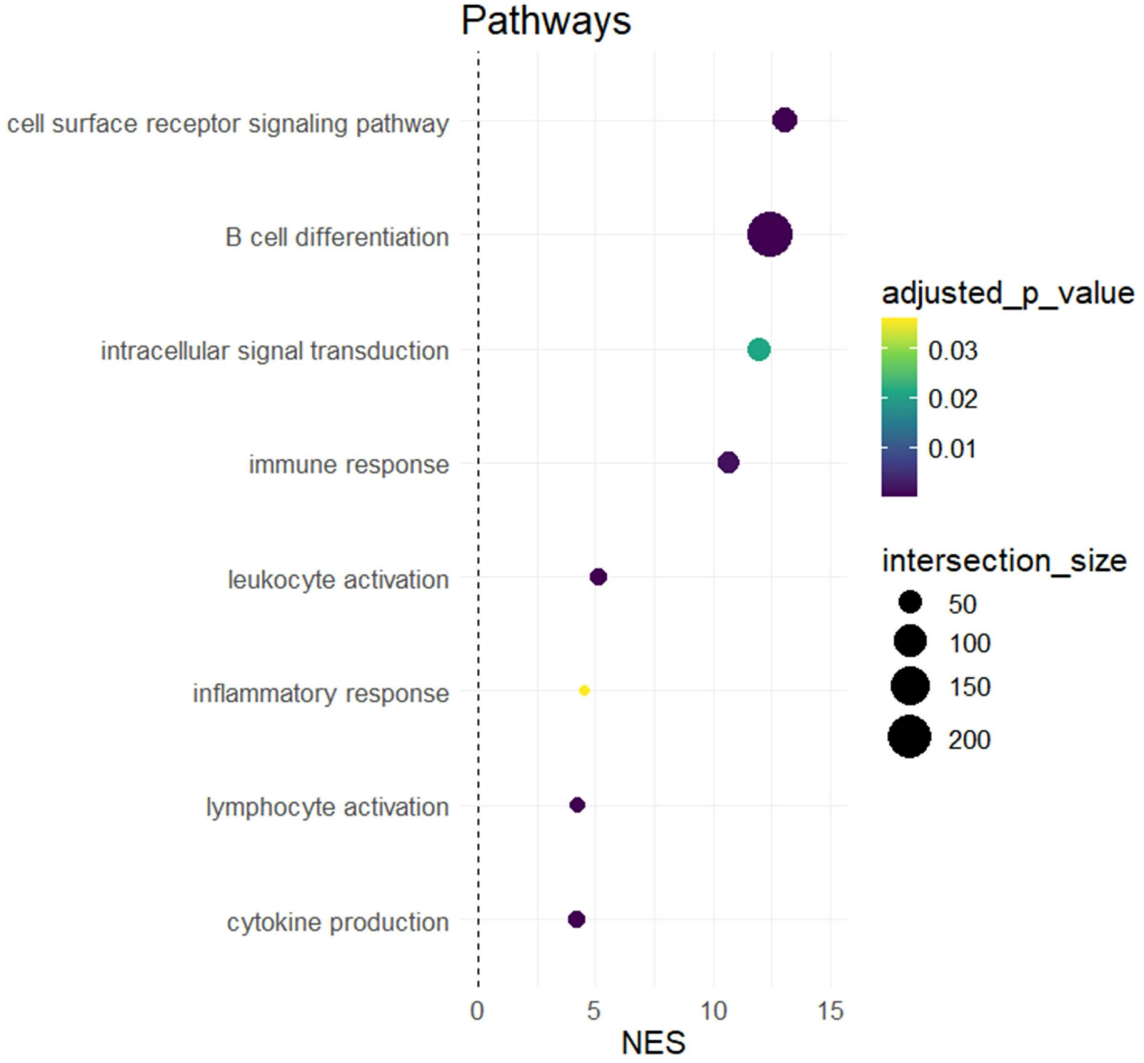
Pathway analysis of genes mapped to by SNPs overrepresented in early repleters (ER). The figure shows the results of a hypergeometric analysis indicating enrichment for pathways related to immune system processes and B cell differentiation. Colors represent adjusted p-values, and the size of the dots represents the number of genes included in the noted pathway.

To investigate potential functional relationships between SNPs that are more common in ER, we completed a pathway analysis of SNPs with known variant effects on mapped genes (**Figure 6**).

Using g:Profiler, we assessed pathway enrichment of genes affected by SNPs overrepresented in ER patients. SNPs overrepresented in ERs mapped to genes that were enriched in pathways related to cell surface receptor signaling (adjusted p =5.94E-05, normalized enrichment score (NES) = 13.1), intracellular signal transduction (adjusted p =0.02, NES =12.0), immune response (adjusted p =0.002, NES =10.7), leukocyte activation (adjusted p-value=3.06E-6, NES=5.08), inflammatory response (adjusted p=0.04, NES=4.5), lymphocyte activation (adjusted p=1.7E-5, NES=4.2), and cytokine production (adjusted p=1.46E-07, NES=4.2) (**Figure 6**). We also specifically assessed whether there was an overrepresentation in ER patients of SNPs of genes known to play roles in B cell differentiation (15–18). Our analysis identified an overrepresentation of 228 genes with known B cell differentiation-related function (adjusted p-value=1.9E-262, NES=12.5). Notably, these genes included BACH2, IRF1, and other key transcription factors critical for B cell development, as well as TNFSF13B, the gene encoding BAFF itself.

The genes that map to known functional pathways related to SNPs overrepresented in ER are listed in **Supplementary Table S3**.

### 3.6 SNPs associated with the most rapid B-cell repletion

Within our group of 18 ER without ADA, we identified a subgroup of 7 patients, whom we termed “super-repleters,” as each displayed circulating B cell levels of >2% at 4 months after anti-CD20 therapy. In the seven super-repleters, 45 SNPs were significantly overrepresented compared to the other ER patients (Fisher’s exact test, p < 0.05), and the higher minor allele frequency (MAF) in these super-repleters was also significantly overrepresented in ER compared to NR, including genes enriched for immune receptor activity; IL17RE, IL2RB, IL17RD, IL1R1, IL23R, and IL1RL2 (**Supplementary Table S4**) that are all related to the inflammatory pathway.

We wondered whether differences in genetic inheritance in ER could contribute to variations in levels of soluble CD40L (sCD40L) that arise via cleavage from the lymphocyte membrane. We, therefore, dichotomized the ER patients based on serum sCD40L levels: those with high sCD40L (n = 4) and those with low sCD40L (n = 14), with high levels defined as greater than the mean + SEM, as shown in **Figure 2**. For the ERs with these raised levels of serum sCD40L, we identified 27 SNPs that were significantly overrepresented compared to the representation in ER patients with low sCD40L (Fisher’s exact test p < 0.05). In the high sCD40L ER patients, the genes with higher MAF were also overrepresented in NR vs. ER comparisons, and this set included genes involved in interleukin-12-related signaling: PLCB1, IL12RB2, and STAT4 (**Supplementary Table S5**). Compared to the low sCD40L ER patients, the high sCD40L ER patients also had an overrepresentation of an SNP associated with an intron variant in the transcription factor, Bach1, which regulates B cell development and differentiation (21).

Alleles over-represented in ER patients were also associated with MS disease risk.

In our cohort, we also examined genes previously identified as non-MHC risk alleles for MS in AA patients (22). Among the assessed rsIDs, significant associations were observed for seven SNPs, previously described by Isobe et al. (22), which were overrepresented in ER patients compared to NR in our cohort. These genes include WWOX, DDAH1, STAT3, CLEC16A, IL2RA, BACH2, and PHGDH. Notably, rs12149527, which has been associated with altered transcript half-life of the gene WWOX, was previously identified by Isobe et al. (22) as an MS genetic susceptibility factor in AA individuals. In our study, WWOX was also associated with ER.

Such effects on transcript levels can lead to altered expression, potentially impacting the gene’s function as a susceptibility gene for MS (**Table 3**). Hence, mechanisms predisposing AA individuals to develop MS may overlap with mechanisms that are responsible for early B cell repopulation, suggesting that pathologic B cell autoimmunity may also be intertwined with the drivers of faster B cell repopulation after anti-CD20 infusion.

**Table 3:** SNPs overrepresented in Early Repleters (ERs) compared to Normal Repleters (NRs) and the associated with MS risk genes.

RsID	Name	Chrom	MAF ER	MAF NR	OR Homoz vs. germ	OR Heteroz vs. germ	pvalue
rs8099409	TMEM241	18	0.75	0.30	84.31 (5.21, 1363)	5.39 (0.61,47)	0.0003
rs7228616	TMEM241	18	0.42	0.17	6.03 (0.49,74)	5.74 (1.41,23)	0.017
rs76709352	TMEM241	18	0.33	0.04	32.69 (0.05,22389)	14.29 (2.58,79)	0.001
rs6507572	TMEM241	18	0.56	0.33	6.99 (0.87,56)	7.03 (1.27,39)	0.044
rs7239133	TMEM241	18	0.47	0.13	115.84 (0.18,73899)	11.85 (2.53,56)	0.0005
rs2304301	TMEM241,CABLES1	18	0.11	0.00	1.64 (0.0002,10764)	67.17 (0.12,36875)	0.030
rs10502437	TMEM241	18	0.19	0.13	0.08 (0.0001,49)	5.82 (1.05,32)	0.030
rs12608315	TMEM241	18	0.19	0.13	0.08 (0.0001,49)	5.82 (1.05,32)	0.030
rs8093392	TMEM241	18	0.19	0.02	1.99 (0.0003,13142)	12.85 (1.52,109)	0.013
rs370159098	BACH2	6	0.64	0.28	19.48 (2.33,163)	5.76 (1.02,32)	0.011
rs7178117	SMAD3	15	0.64	0.41	12.11 (1.05,139)	0.68 (0.13,3.6)	0.008
rs12912010	SMAD3	15	0.22	0.02	21.9 (0.03,14768)	11.04 (1.28,95)	0.019
rs12912045	SMAD3	15	0.22	0.09	0.18 (0.0002,122)	7.68 (1.41,42)	0.018
rs12913547	SMAD3	15	0.22	0.07	1.99 (0.0003,13175)	5.2 (1.15,24)	0.036
rs35251008	SMAD3	15	0.22	0.09	0.18 (0.0002,122)	7.68 (1.4,42)	0.018
rs4534865	CLEC16A	16	0.61	0.54	3.62 (0.37,35)	11 (1.2,100)	0.042
rs7203459	CLEC16A	16	0.31	0.07	27.3 (0.04,18612)	7.28 (1.58,33)	0.008
rs9926590	CLEC16A	16	0.17	0.02	1.83 (0.0002,12037)	10.13 (1.18,87)	0.031
rs16957952	CLEC16A	16	0.11	0.00	1.64 (0.0002,10764)	67.17 (0.12,36875)	0.030
rs12510546	TIFA	4	0.61	0.24	27.28 (2.58,289)	3.76 (0.79,18)	0.005
rs12942547	STAT3	17	0.56	0.41	5.03 (0.44,57)	12.82 (1.5,110)	0.023
rs1905340	STAT3	17	0.56	0.39	6.25 (0.53,74)	11.55 (1.36,98)	0.040
rs35314169	STAT3	17	0.56	0.39	6.25 (0.53,74)	11.55 (1.36,98)	0.040
rs3785898	STAT3	17	0.56	0.39	6.25 (0.53,74)	11.55 (1.36,98)	0.040
rs8068748	STAT3	17	0.56	0.41	5.03 (0.44,57)	12.82 (1.5,110)	0.023
rs957970	STAT3	17	0.56	0.41	5.03 (0.44,57)	12.82 (1.5,110)	0.023
rs9891119	STAT3	17	0.56	0.41	5.03 (0.44,57)	12.82 (1.5,110)	0.023
rs2582869	TNFSF13B	13	0.50	0.28	4.23 (0.57,31)	7.2 (1.53,34)	0.024
rs35154155	SH2D4B	10	0.50	0.13	16.45 (1.37,198)	17.23 (3.33,89)	0.0002
rs7895413	SH2D4B	10	0.22	0.02	21.9 (0.03,14768)	11.04 (1.28,95)	0.019
rs72805746	SH2D4B	10	0.17	0.02	1.83 (0.0002,12037)	10.13 (1.18,87)	0.031
rs4657040	FCGR2A	1	0.50	0.20	13.73 (1.39,136)	3.38 (0.81,14)	0.026
rs809356	IL2RA	10	0.47	0.22	4.87 (0.5,47)	10.46 (2.2,50)	0.004
rs4625363	IL2RA	10	0.25	0.09	2.1 (0.0003,13937)	4.66 (1.14,19)	0.043
rs6662887	TGFBR3	1	0.47	0.13	12.44 (1.1,141)	11.95 (2.5,57)	0.001
rs6693438	TGFBR3	1	0.39	0.13	5.66 (0.47,68)	7.31 (1.68,32)	0.009
rs1018159	WWOX	16	0.44	0.26	2.21 (0.16,30)	7.36 (1.63,33)	0.011
rs4888826	WWOX	16	0.42	0.30	0.74 (0.07,8)	10.99 (2.3,52)	0.002
rs4887974	WWOX	16	0.39	0.30	0.07 (0.0001,39)	11.83 (2.5,56)	0.0002
rs12149527	WWOX	16	0.19	0.04	1.9 (0.0002,12554)	6.43 (1.17,35)	0.028
rs1421627	BANK1	4	0.42	0.26	1.67 (0.13,22)	5.17 (1.26,21)	0.048
rs35388091	BANK1	4	0.42	0.26	1.67 (0.13,22)	5.17 (1.26,21)	0.048
rs2851320	BANK1	4	0.11	0.00	1.64 (0.0002,10764)	67.17 (0.12,36875)	0.030
rs2851334	BANK1	4	0.11	0.00	1.64 (0.0002,10764)	67.17 (0.12,36875)	0.030
rs34166099	BANK1	4	0.11	0.00	1.64 (0.0002,10764)	67.17 (0.12,36875)	0.030
rs1153285	BACH1	21	0.19	0.04	1.9 (0.0002,12554)	6.43 (1.17,35)	0.028
rs1236481	BACH1	21	0.19	0.02	1.99 (0.0003,13143)	12.85 (1.52,109)	0.013
rs12129013	DDAH1,BCL10-AS1	1	0.25	0.11	0.19 (0.0002,130)	6.16 (1.36,28)	0.016
rs974874	DDAH1	1	0.22	0.04	2.03 (0.0003,13823)	8.06 (1.48,44)	0.012
rs12482248	RUNX1	21	0.17	0.02	1.83 (0.0002,12037)	10.13 (1.18,87)	0.031
rs3213159	E2F1	20	0.11	0.00	1.64 (0.0002,10765)	67.17 (0.12,36875)	0.030
rs41429347	STAT1	2	0.11	0.00	1.64 (0.0002,10765)	67.17 (0.12,36875)	0.030

Data are included in the columns: rsID (reference SNP cluster ID), Chr (chromosome), Mapped Gene, MAF ER (mean allele frequency in ER), MAF NR (mean allele frequency in NR), p-value, OR Homozygotic vs. Germline (odds ratio for homozygous vs. germline; OR; 95% confidence interval), OR Heterozygous vs. Germline (odds ratio for heterozygous vs. germline; OR; 95% confidence interval). Genetic variations that are significantly overrepresented in ER and their potential impact on B cell repopulation dynamics are highlighted. These are the top SNPs and mapped genes of interest identified for future studies of MS-risk alleles for early B cell repletion post-CD20 targeted therapy. **Supplementary Table S2** includes variant effects of rsIDs noted.

## 4 Discussion

Our investigations confirmed that amongst AA patients with neurologist-diagnosed MS, there is a distinct subpopulation, here termed ER, exhibiting a time-shortened B cell depletion response to standard semi-annual treatment dosing of anti-CD20 monoclonal antibodies. In both ER and NR, the repletion pattern was dominated by early B cell subsets (transitional and immature/naïve B cells), likely emerging after being newly generated in the bone marrow.

We assessed anti-CD20 drug concentrations and the presence of anti-drug antibodies. To subvert the development of anti-drug antibody responses, therapeutic anti-CD20 antibodies, including OCR, were engineered to be more human-like, yet there remain molecular features that are foreign to the human immune system. Among the 43 AA MS patients studied, we identified early repleters that included two patients with high concentrations of anti-OCR antibodies (4.6%), which was higher than the 0.4% prevalence reported in a clinical randomized trial with patients of diverse ethnicities and races (6). Yet, our population was selected in part based on their early B cell repopulation in prior treatment cycles. Predictably, the host anti-OCR antibodies in two of our ER patients appeared to functionally neutralize and/or enhance OCR clearance, which correlated with early post-infusion B cell repletion. The impact of host anti-OCR antibodies on drug clearance and B cell repopulation highlights the need for further assessment of OCR’s effectiveness in relation to this immune response in larger studies, especially regarding the influence of anti-idiotypic antibodies (23). From a classical perspective, anti-idiotypic antibodies may represent molecular mimicry of the CD20 antigen. This structural resemblance in theory can enable interactions with components of the immune network, thereby potentially enhancing the effects of the infused anti-CD20 antibody by bolstering CD20-directed immunity. Conversely, as observed in the two patients in our study who developed anti-drug antibodies, it may diminish the antibody’s efficacy through mechanisms such as inducing neutralizing antibodies or altering drug disposition (24). This network’s role in autoimmunity and its regulation is supported by both theoretical models and experimental data (24, 25). Furthermore, the clonal expansion of B cells, as observed in other autoimmune conditions like pediatric anti-NMDAR encephalitis, underscores the importance of understanding B cell dynamics in MS (25). In MS, B cells contribute to disease progression through clonal expansion of pathogenic B cells that can drive cytokine production, antigen presentation, and antibody synthesis, with different B cell subsets exerting pro- or anti-inflammatory effects (26). The humoral immune responses, including the production of anti-drug antibodies, may vary among different ethnic groups, potentially contributing to the observed disparities in MS severity (27). Whether patients of African Ancestry are more likely to develop anti-drug antibodies is a question that warrants further study.

Multiple lines of evidence suggest that individuals of African descent can have inherent differences in B cell repopulation kinetics that could influence their disease course in MS and other autoimmune diseases. AA patients with MS are reported to have a higher representation of B cells and plasmablasts in their cerebrospinal fluid (28), and more commonly exhibit oligoclonal bands than patients of other race/ethnic groups (27, 29). Indeed, AA patients with systemic lupus erythematosus display higher serum BAFF levels and a greater number of circulating activated B cells (30–32). Furthermore, even among healthy individuals, AA patients tend to demonstrate heightened neutralizing antibody responses to influenza vaccination (33, 34).

In our patients, we observed an appropriate reciprocal decrease in serum BAFF levels concurrent with the period of peripheral B cellreplenishment. In MS patients, the differences in BAFF levels in ER and NR thus appear to be a consequence. rather than the cause of early repletion. While lower BAFF levels in sera may, in part, be secondary to increased B cell repletion in ER patients, this does not rule out the role of SNP variations on BAFF function on the potential pathogenic roles of B-cells in autoimmunity. TNFSF13B, which encodes the BAFF cytokine, plays a central role in peripheral B-cell survival and maturation. Notably, an intron variant and a non-coding transcript variant SNP (rs2582869), which potentially impact TNFSF13B transcript half-life (encoding BAFF), were overrepresented in ER patients, and this same SNP is also associated with the B cell cancer, non-Hodgkins lymphoma (NHL) (35).

Even when serum BAFF levels are unremarkable, cis-based cell signaling can still have enhanced survival effects on a B cell. Although the exact impact of rs2582869 on BAFF expression has not been studied, the SNP rs9514827 in the promoter of BAFF (-871C->T) has been associated with increased BAFF transcription (36). Notably, rs2582869 and rs9514827 are in the same genomic region, suggesting that genetic variations in this area may both have significant effects on BAFF function and resultant B cell activity. A full summary of all SNPs studied related to BAFF and BAFF-R are included in **Supplementary Table S6**. We also found overrepresentation of other SNPs relevant to immune pathways, including those with known effects on the following genes: IL4, STAT3, and NOD2 (inflammatory response), IL2RA, CD226, CCR6 (leukocyte activation), EBF1, BANK1, and BACH2 (B cell differentiation). Additionally, SNPs that map to genes such as TMEM241, SMAD3, TIFA, STAT3, SH2D4B, and TGFBR3 were identified in our study, which may contribute to inflammatory responses in MS patients. A more in-depth understanding of these pathways could guide future research in developing personalized therapeutic strategies for MS patients with higher genetic polymorphism.

We also found that the SNP rs465704, which maps to the FCGR2A gene, is overrepresented in ER MS patients. This SNP is classified as both an NMD (nonsense-mediated decay) transcript variant and an intron variant (**Supplementary Table S2**), which may affect the half-life of the transcript. FCGR2A polymorphisms have been shown to affect the pharmacogenetics of rituximab by altering the cytotoxic function of macrophages and natural killer cells, thereby impacting the efficacy of monoclonal antibody therapies. Antibody-dependent Cellular cytotoxicity (ADCC) plays a crucial role in the clinical effectiveness of rituximab in a range of disease states (37). Investigations in other autoimmune diseases, including NMO (38), have identified polymorphisms in immunoglobulin Fc receptors that are linked to impaired anti-CD20-mediated B cell depletion and reduced therapeutic benefits (38).

This study has limitations, including that it was from a single center with a limited number of patients studied. In this context, traditional methods to evaluate false discovery rates (FDR) may at times be overly conservative, leading to Type II errors (i.e., false negatives). Therefore, we utilized a multi-step approach that considered multiple statistical methods and only defined as SNPs of interest those that met at least two of the previously determined criteria of significance (**Supplementary Figure S2**). This study did not investigate the impact of SNPs on transcription levels. Instead, we concentrated on identifying genetic polymorphisms in our cohort that are crucial for consideration in larger future studies. Our study did not specify the roles that these genetic polymorphisms may play in immune cell pathways related to early repletion in AA patients with MS. Future studies will focus on integrating single-cell or cell-type specific bulk gene expression with at-risk genetic polymorphisms as clinical variables to better stratify our MS patient cohort and profile immune cell transcriptomes, particularly examining gene expression changes in B cells relative to other immune cells. Furthermore, the existing evidence showing an overrepresentation of SNPs of interest in affected AA patients necessitates independent validation in a larger group of patients receiving anti-CD20 therapy across various institutions.

After the drug wears off replenishment with early B cell subsets is generally associated with a good short-term prognosis as these subsets of B cells are not known to have pathogenic roles in MS (37). However, there are several caveats to this finding: the number of ER AA MS patients was small; the period of therapy was about 5 years on average, which does not allow us to comment on longer-term outcomes. Recent studies, in which the time between anti-CD20 infusions was extended, have not translated into higher rates of MS lesions on MRI or clinical relapses (39–44). Yet, disease progression independent of relapses and MRI lesions may now be the main driver of disease progression in MS patients (44). In fact, in a recent cohort of 148 patients, sub-optimal B-cell depletion was the strongest predictor of progression (45). Whether AA patients with early B cell repletion, the biology of the ER group that we have here identified, have more frequent manifestations of clinical relapse and disability outcomes warrants close investigation.

Few studies have focused on the clinical, immunologic, or pathologic features of MS in AA patients. Our study on MS patients of African descent was made possible by the large cohort of AA patients with MS who receive their care at NYU MS Comprehensive Care Center – 20% of the individuals in our MS center self-identify as AA. Social and economic factors are common confounders in studies of under-represented groups and likely contribute to worse outcomes seen in AA patients with MS (46). As our participants were receiving care in a specialized tertiary center with uniform treatment protocols, this may reduce, but cannot eliminate, such concerns. While acknowledging the importance of socio-economic factors, our analyses suggest that immunogenetic factors may be contributory drivers in some patients.

Our study highlights several gaps in the literature. There have been no prior pharmacogenetic studies that specifically investigated the relationship between genetic variations and biological, clinical, and radiographic outcomes of anti-CD20 therapy in MS patients (37). There is increasing clinical interest that deeper B cell depletion, may convey greater clinical benefits (i.e., lower risk of progression independent of relapses) (46) and this may require the use of B cell targeted Chimeric Antigen Receptor (CAR) expressing T cell therapy, and here we have shown there may be a biologic basis for concern regarding treatment efficacy in a subset of AA early repleters. Our study also raises an important question of whether the differences in B cell repopulation kinetics documented in individuals of African descent could contribute to differences in clinical disease course in AA patients with MS.

In conclusion, our study in AA patients found a subset of the ER patients in which, a B cell extrinsic factor (i.e., the induction of anti-drug antibodies) was responsible for earlier B-cell repopulation. In other AA patients displaying the ER phenotype, we identified an associated inheritance of SNPs linked to pathways involved in the inflammatory response, in leukocyte activation, and in B cell activation and differentiation.

As there is now a trend in clinical practice to extend the interval between dosing to minimize infectious complications in anti-CD20 treated MS patients, it is important to understand how this strategy will affect patients. The prospective identification of MS patients especially amongst those of African descent, with these SNPs could potentially be used for individualizing the timing interval between courses of CD20-depleting therapy to minimize the risk of disease progression, in those who have more rapid B cell repletion. Hence, future pharmacogenetic studies of larger cohorts, that integrate studies of SNPs affecting ADCC and B cell survival, could also help to further optimize therapeutic outcomes.

## Data Availability

The genetic polymorphism data supporting the findings of this study are openly available in the Gene Expression Omnibus (GEO) database under the accession number GSE297571.
