# Correction: B cell-extrinsic and intrinsic factors linked to early immune repletion after anti-CD20 therapy in patients with multiple sclerosis of African ancestry

**DOI:** 10.3389/fimmu.2025.1649409

**Published:** 2025-07-22

**Authors:** Gregg J. Silverman, Abhimanyu N. Amarnani, Arnaldo A. Arbini, Angie Kim, Hannah Kopinsky, David Fenyo, Ilya Kister

**Affiliations:** ^1^ Department of Medicine, Division of Rheumatology, NYU Grossman School of Medicine, New York, NY, United States; ^2^ Department of Pathology, NYU Grossman School of Medicine, New York, NY, United States; ^3^ Department of Neurology, NYU Grossman School of Medicine, New York, NY, United States; ^4^ Department of Biochemistry and Molecular Pharmacology, NYU Grossman School of Medicine, New York, NY, United States

**Keywords:** Ocrelizumab, multiple sclerosis, rituximab, CD20, African ancestry, B-cell repletion, genetics, B-cell activating factor

Author “Arnaldo A. Arbini” was erroneously spelled as “Arnaldo A. Armini”.

A correction has been made to the **Abstract**, first paragraph (Introduction)

Error:

“In this study, we explored the immunologic and genetic factors, with, serum drug monitoring that may contribute to a faster rate of B-cell repletion that follows during recovery from treatment with anti-CD20 antibody.”

Corrected sentence:

“In this study, we explored the immunologic and genetic factors, with serum drug monitoring, that may contribute to a faster rate of B-cell repletion that follows during recovery from treatment with anti-CD20 antibody.”

A correction has been made to the **Introduction**, first paragraph (after heading “1 Introduction”)

Error:

“Suspicion of the potential roles of B-cells in MS was first suggested based on oligoclonal immunoglobulin bands in the CSF of MS patients (4, 5) and later confirmed by on the clinical efficacy of B-cell-depleting therapies in suppressing relapses and new MRI lesions in MS (6, 7).”

Corrected sentence:

“Suspicion of the potential roles of B-cells in MS was first suggested based on oligoclonal immunoglobulin bands in the CSF of MS patients (4, 5) and later confirmed by the clinical efficacy of B-cell-depleting therapies in suppressing relapses and new MRI lesions in MS (6, 7).”

A correction has been made to the **Materials and methods**, *Flow cytometric analyses (Section 2.3)*, second paragraph

Error:

“Cells were washed fixed, and data was acquired on a 5-laser (355/405/488/561/637 nm) SONY ID7000 Spectral analyzer.”

Corrected sentence:

“Cells were washed, fixed, and data were acquired on a 5-laser (355/405/488/561/637 nm) SONY ID7000 Spectral analyzer.”

A correction has been made to the **Materials and methods**, *Statistical analyses of genetic SNP (Section 2.5)*, second paragraph

Error:

“Additionally, among the significantly overrepresented SNPs (Fisher’s exact test, p < 0.005) with a positive mean allele frequency in ERs compared to NRs, we identified 317 SNPs of interest that had a mean allele frequency of for NRs but an allele frequency of at least 2 in ERs (Supplementary Table S2).”

Corrected sentence:

“Additionally, among the significantly overrepresented SNPs (Fisher’s exact test, p < 0.005) with a positive mean allele frequency in ERs compared to NRs, we identified 317 SNPs of interest that had a mean allele frequency for NRs but an allele frequency of at least 2 in ERs (Supplementary Table S2).”

A correction has been made to the **Discussion**, fifth paragraph (after heading “4 Discussion”)

Error:

“In our patients, we observed an appropriate reciprocal decrease in serum BAFF levels concurrent with the period of peripheral B cell replenishment. In MS patients, the differences in BAFF levels in ER and NR thus appear to be a conseque”

Corrected sentence:

“In our patients, we observed an appropriate reciprocal decrease in serum BAFF levels concurrent with the period of peripheral B cell replenishment. In MS patients, the differences in BAFF levels in ER and NR thus appear to be a consequence.”

The original version of this article has been updated.

