# Supplementary material for: B cell-extrinsic and intrinsic factors linked to early immune repletion after anti-CD20 therapy in patients with multiple sclerosis of African ancestry
Source: Front Immunol. 2025 Jun 10;16:1590165. doi: 10.3389/fimmu.2025.1590165 (PMC12185503; doi:10.3389/fimmu.2025.1590165)
Supplement: Supplementary file 1 [file DataSheet1.pdf]

Supplementary Material

B cell-extrinsic and intrinsic factors linked to early immune repletion after anti-CD20 therapy in patients with Multiple Sclerosis of African Ancestry

Gregg J. Silverman[1*], Abhimanyu N. Amarnani[1], Arnaldo A. Armini[2], Angie Kim[3], Hannah Kopinsky[3], David Fenyo[4], Ilya Kister[3]

Affiliations:

1 Department of Medicine, Division of Rheumatology, NYU Grossman School of Medicine

2 Department of Pathology, NYU Grossman School of Medicine

3 Department of Neurology, NYU Grossman School of Medicine

4 Department of Biochemistry and Molecular Pharmacology, NYU Grossman School of Medicine

* For correspondence Gregg.Silverman@nyulangone.org

Supplementary Figures and Tables

1.1 Supplementary Tables

Supplemental Table 1.

Marker	Fluorochrome	Clone	Vendor
**Live/Dead Fixable Blue**	BUV496	N/A	ThermoFisher
**CD19**	BUV496	SJ25C1	BD Biosciences
**CD25**	BUV563	2A3	BD Biosciences
**IgD**	BUV737	IA6-2	BD Biosciences
**CD14**	BUV805	M5E2	BD Biosciences
**CD16**	BV480	3G8	BD Biosciences
**CD4**	SparkViolet-538	SK3	Biolegend
**CD56**	BV570	5.1H11	Biolegend
**CD27**	BV711	O323	Biolegend
**CXCR5**	BB515	RF8B2	BD Biosciences
**CD3**	SparkBlue-550	SK7	Biolegend
**PD-1**	BB700	EH12.1	BD Biosciences
**CD8**	PE-Fire640	SK1	Biolegend
**FOXP3**	PE-Cy5.5	PCH101	ThermoFisher
**CD45RA**	SparkNIR-685	HI100	Biolegend
**CD278**	APC-Fire750	C398.4A	Biolegend

Supplemental Table 1. Summary of Flow Cytometry Markers, Fluorophores, and Clones Used for Analysis. This table provides detailed information on the markers, fluorophores, and clones used in the flow cytometry analyses.

Legends for Supplemental Table 2,3,4, 5, and 6 (All separate uploaded .xls files)

Supplemental Table 2. 2,202 Genes that are Overrepresented in Early Repleters (ER) compared to Normal Repleters (NR). The table provides a comprehensive list of genes that are significantly overrepresented in ERs. This table includes the following columns: Gene Symbol, Gene Name, Chr (chromosome), Start Position, End Position, Number of SNPs, and gene variant effects. Variant effects are described according to the Sequence Ontology from g:SNPense. NMD (nonsense-mediated decay) transcript variant refers to a variant leading to nonsense-mediated decay of the transcript, reducing the amount of functional mRNA and protein. A missense variant indicates a single nucleotide change resulting in a different amino acid, which can affect protein function. Non-coding transcript variant denotes a variant in a noncoding transcript. 3' UTR variant describes a variant in the 3' untranslated region. Synonymous variant refers to a variant that does not change the amino acid sequence, usually with minimal effect. Intron variant indicates a variant within an intron. Only those effects are shown which correspond to given rs-codes. The displayed number shows the number of variant effects.

Supplemental Table 3. Summary of Pathway Analysis Genes and Overlapping Genes. This table highlights the genes identified through pathway analysis that are significantly associated with the condition of interest and their overlap with other relevant gene sets.

Supplemental Table 4. Summary of SNPs overrepresented in superrepleter patients compared to other early repleters (ER). Highlights the genetic variations that are significantly overrepresented in superrepleter patients. Variant effects are described, defined by the Sequence Ontology, and ordered by severity from g:SNPense.

Supplemental Table 5. Summary of SNPs overrepresented in patients with high sCD40L compared to other early repleters (ER). Highlights the genetic variations that are significantly overrepresented in patients with high serum volumes of sCD40L compared to other ERs. Variant effects are described, defined by the Sequence Ontology, and ordered by severity from g:SNPense.

Supplemental Table 6. BAFF and BAFF-R related single nucleotide polymorphisms. Summary of all SNPs included in the study analysis relating to the BAFF or the BAFF-R, including the minor allele frequency of these SNPs in early repleters (ER) and normal repleters (NR).

2.1 Supplementary Figures

Supplemental Figure 1. Gating strategy for Flow Cytometry Studies.

A) Gating Strategy of white blood cells, singlets, and live/dead cell distinction

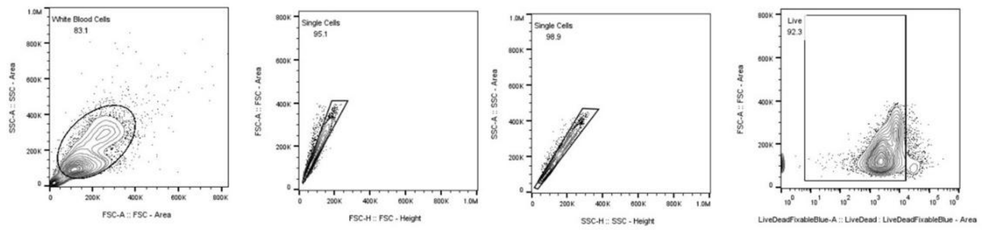

B) Gating Strategy to Define T cells (CD14-CD16-CD3+) and B cells (CD14-CD16-CD19+)

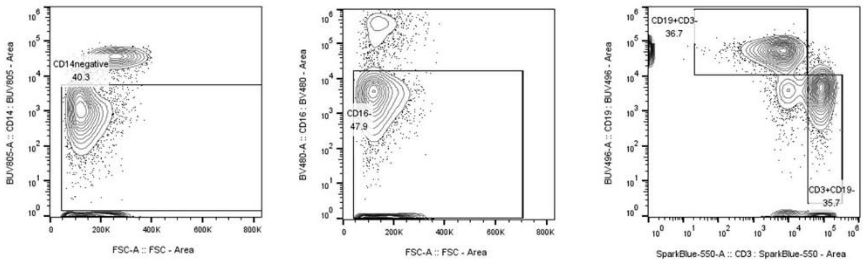

C) Gating Strategy to Define Monocytes (CD3-CD16+CD14- or CD3-CD14+CD16-) and NK cells (CD56dimCD16+)

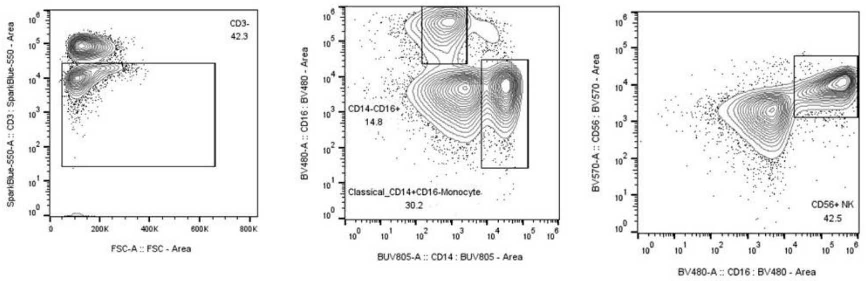

D)

Gating Strategy for B cell subsets (Transitional/Naïve CD27-IgD+, Unswitched Memory CD27+IgD+, Switched CD27+IgD-, and double negative CD27-IgD-)

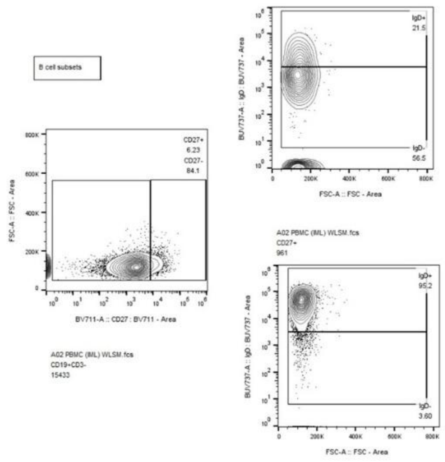

E) Gating Strategy for T cells subsets (CD27+CD45RA+CD4+ Naïve and CD27+CD45RA+CD8+ Naïve)

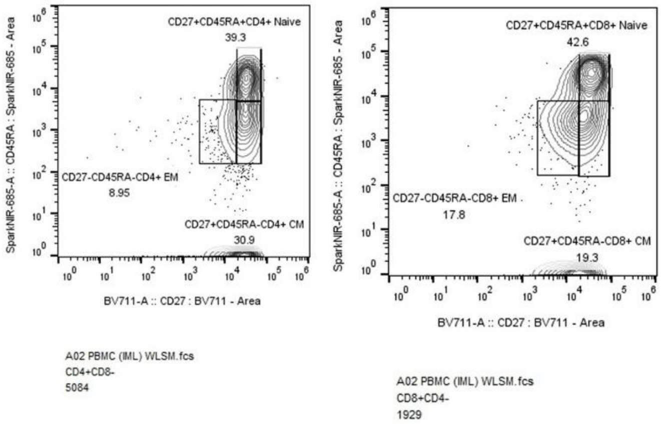

F) Gating Strategy for T cell subsets (T Peripheral Helper Cells PD4+PD1+CXCR5- and Regulatory T cells CD25+ICOS+Foxp3+)

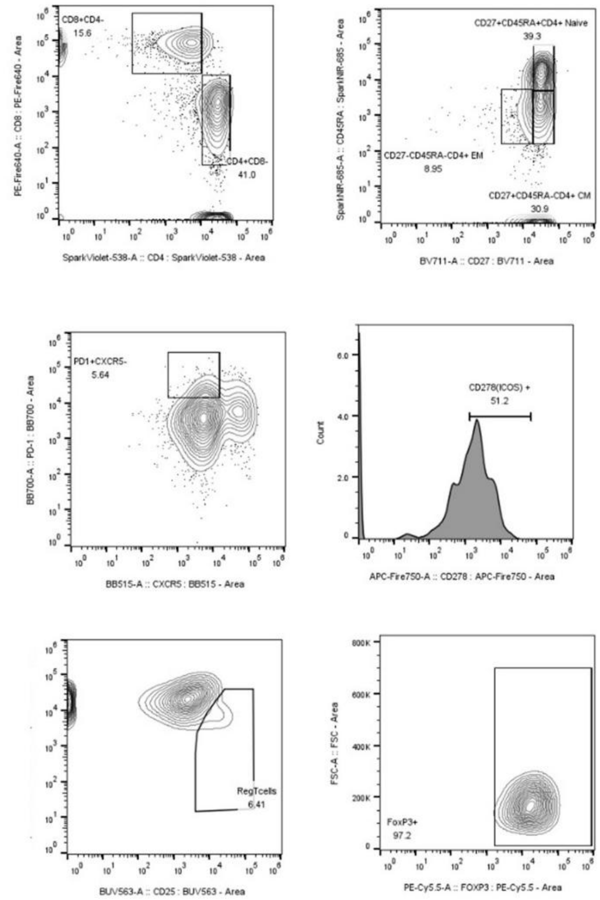

Supplemental Figure 1. Flow cytometry gating strategy used to identify and analyze B cells and various B cell subsets. The gating strategy includes the initial identification of lymphocytes followed by the isolation of CD19+ B cells. Subsequent gates are applied to distinguish between naive B cells, memory B cells, activated naive B cells, and DN2 B cells. Each gating step is shown with representative flow cytometry plots.

Supplemental Figure 2. Flow diagram illustrating the identification of significantly overrepresented SNPs in early repleters (ERs).

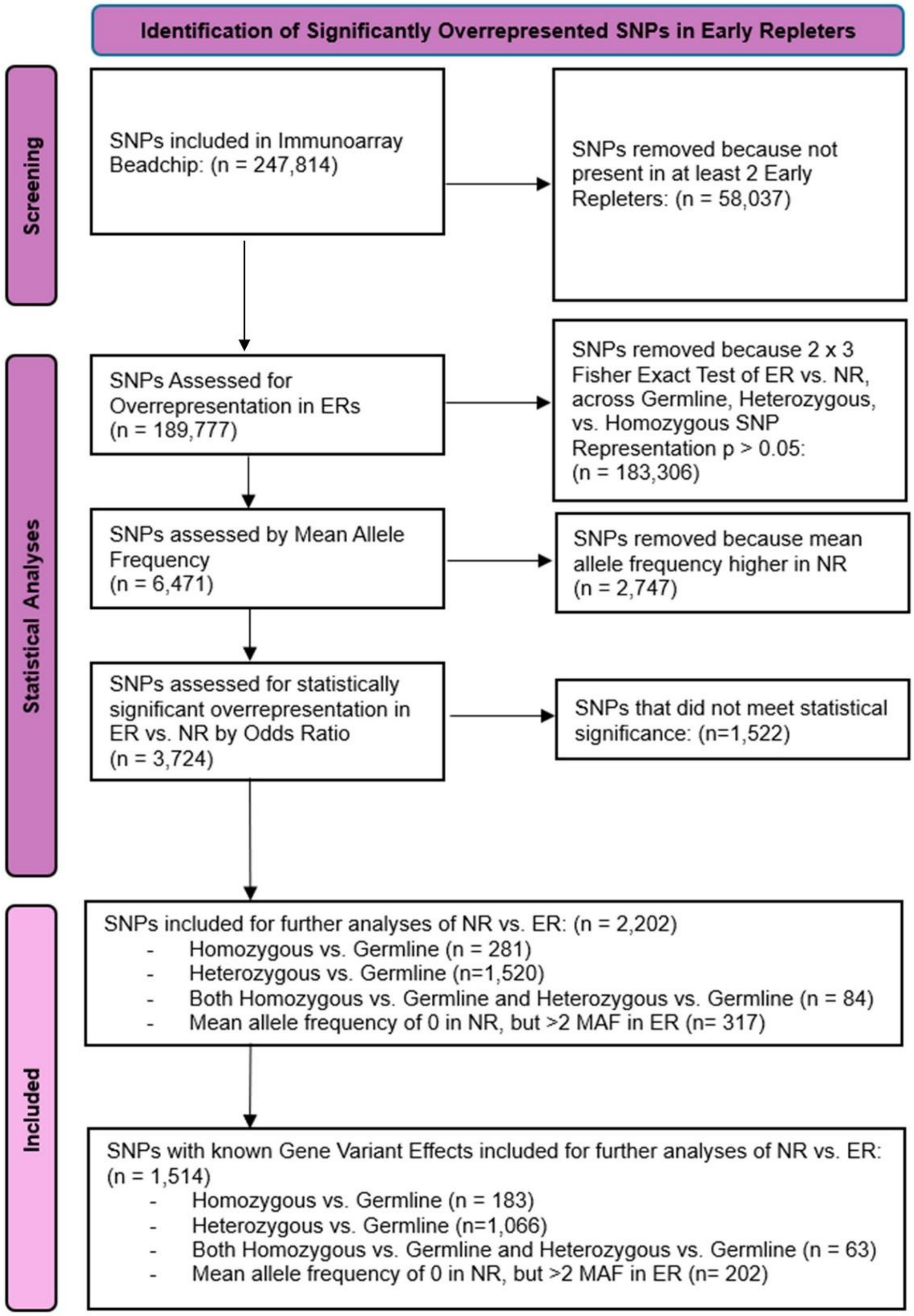

Supplemental Figure 2. The diagram outlines the process starting from the initial screen of SNPs included in the study, followed by multiple statistical assessments to refine the list of SNPs.

Supplemental Figure 3. Clinical and MRI activity in normal repleters (NR) and early repleters (ER).

A)

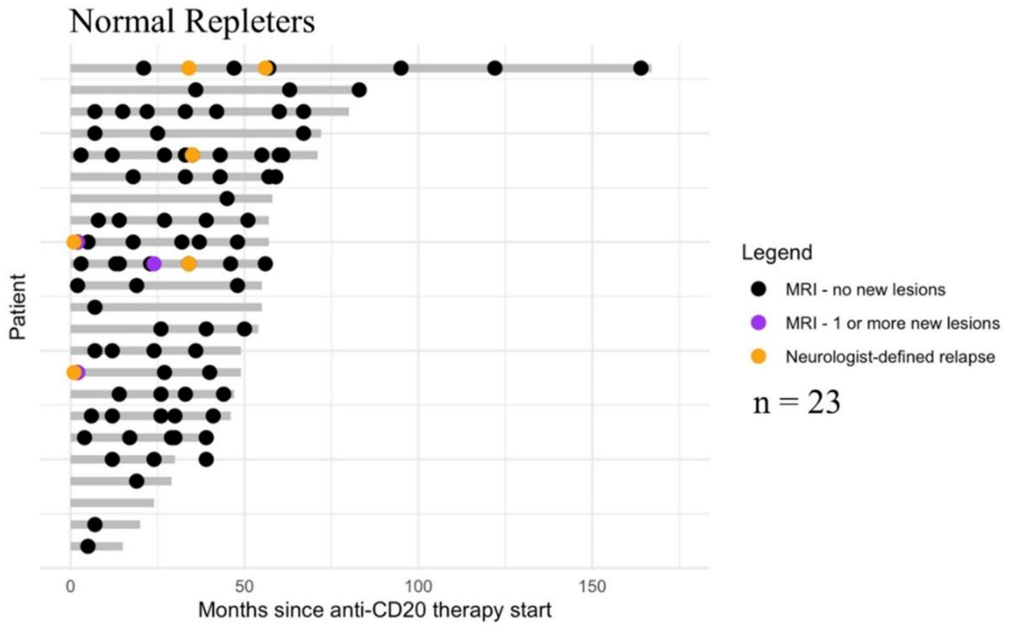

B)

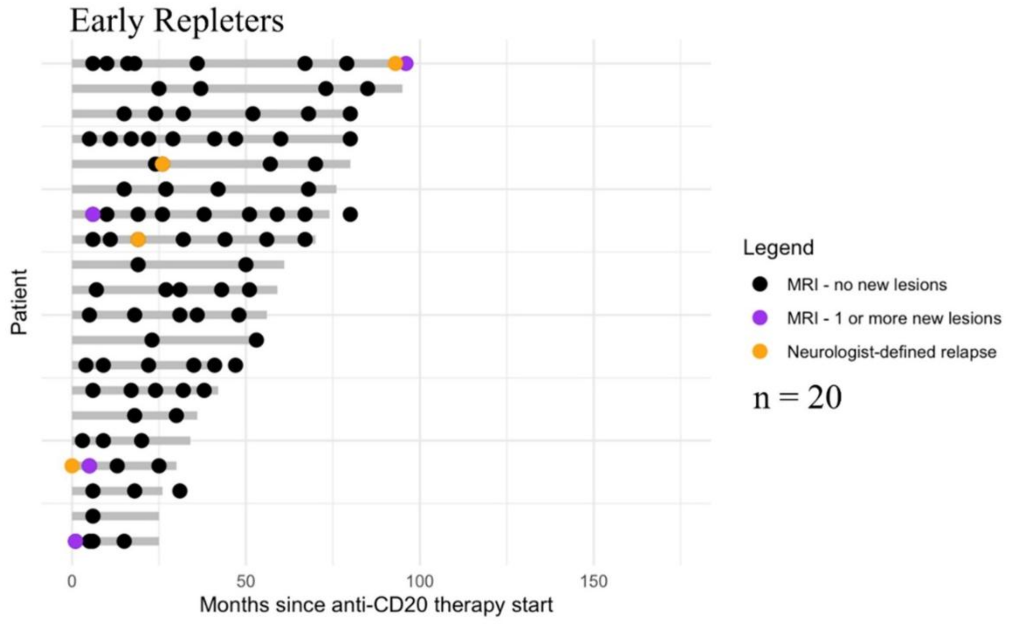

Supplemental Figure 3. Number of MRI evaluations completed per patient assessed (black), with timeline of new MRI lesions (purple) and neurologist-defined relapses (orange).

Supplemental Figure 4. Distribution of serum immunoglobulin levels.

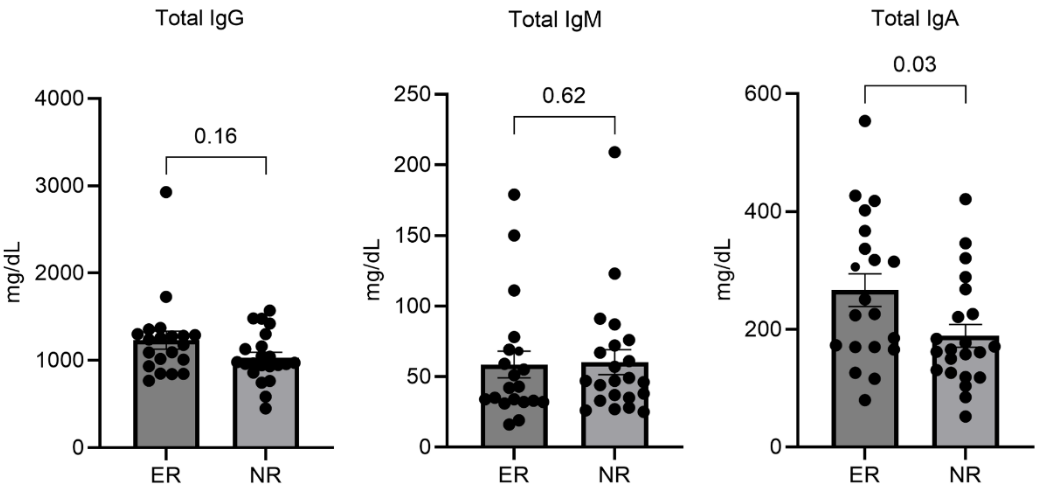

Supplemental Figure 4. Serum immunoglobulin levels of total IgG (left), total IgM (middle), and total IgA (right), in early repleters (ER) vs. normal repleters (NR). Sera data represent one sample per patient. Time since infusion for serum sample collection range from 3-11 months.

Supplemental Figure 5. Correlation of OCR drug concentration and CD19% B cell percentages.

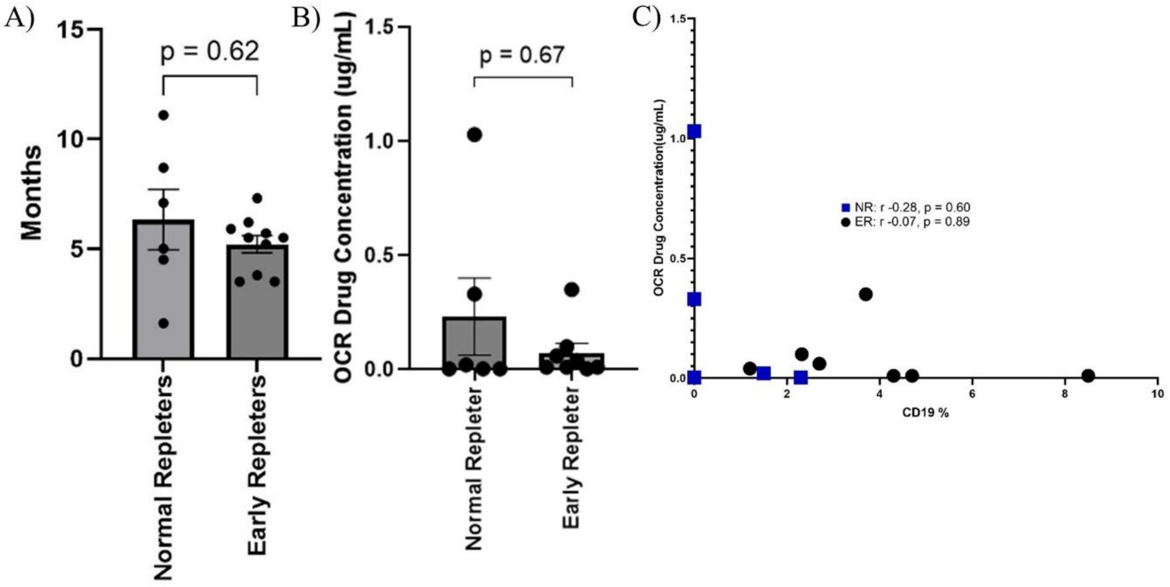

Supplemental Figure 5. (Left) No difference in time since infusion for samples collected for assessment of anti-drug antibodies or ocrelizumab drug concentrations. (Middle) No difference in overall drug concentration between NR (normal repleters) and ER (early repleters) patients studied (n = 6 NRs, n = 8 ERs). (Right) Spearman correlation of CD19% and OCR drug concentration.

Supplemental Figure 6. Correlation of BAFF, BCMA, and sCD40L with time since anti-CD20 infusion

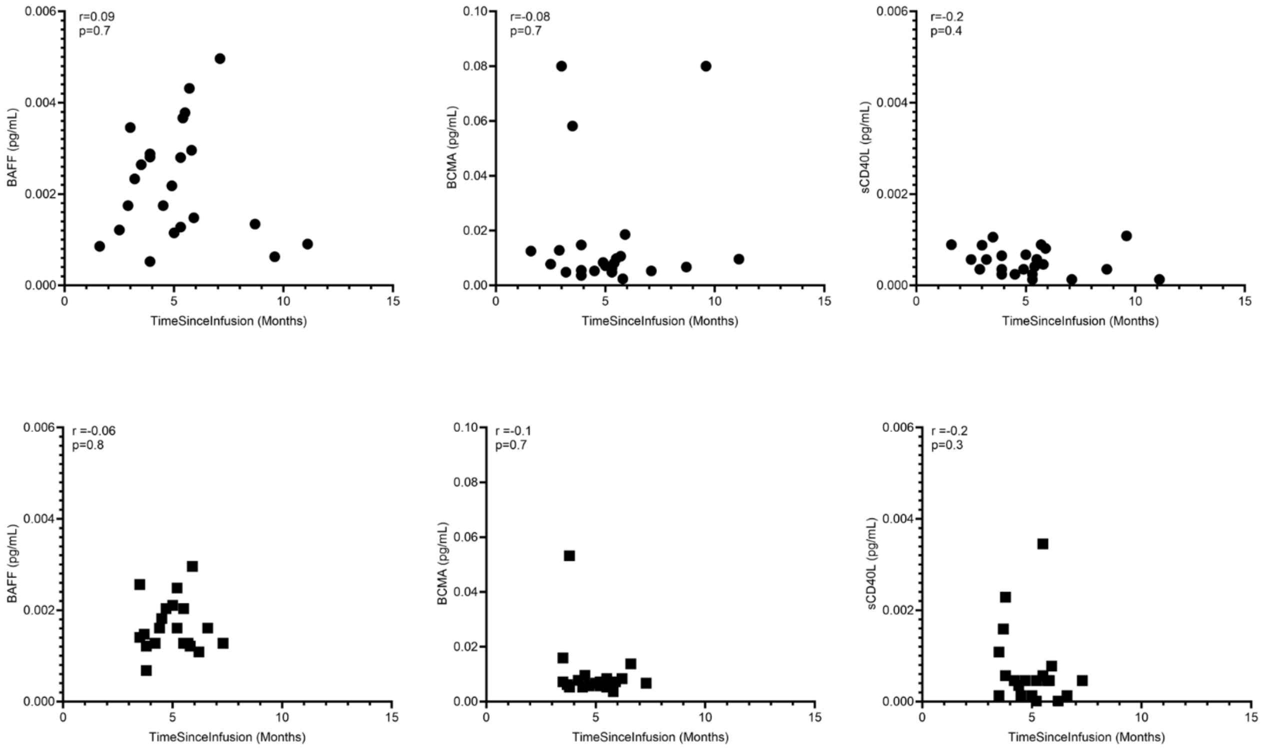

Supplemental Figure 6. Time since infusion correlated with serum levels of B cell activation factor (BAFF) (left), B cell maturation antigen (BCMA) (middle), and soluble CD40L (sCD40L) (right). Statistics represented are non-parametric (spearman r) correlations.

Supplemental Figure 7. Summary of the proportions of immune cell subsets in expression ER and NR participants ratio (ER) and non-responders (NR) patients.

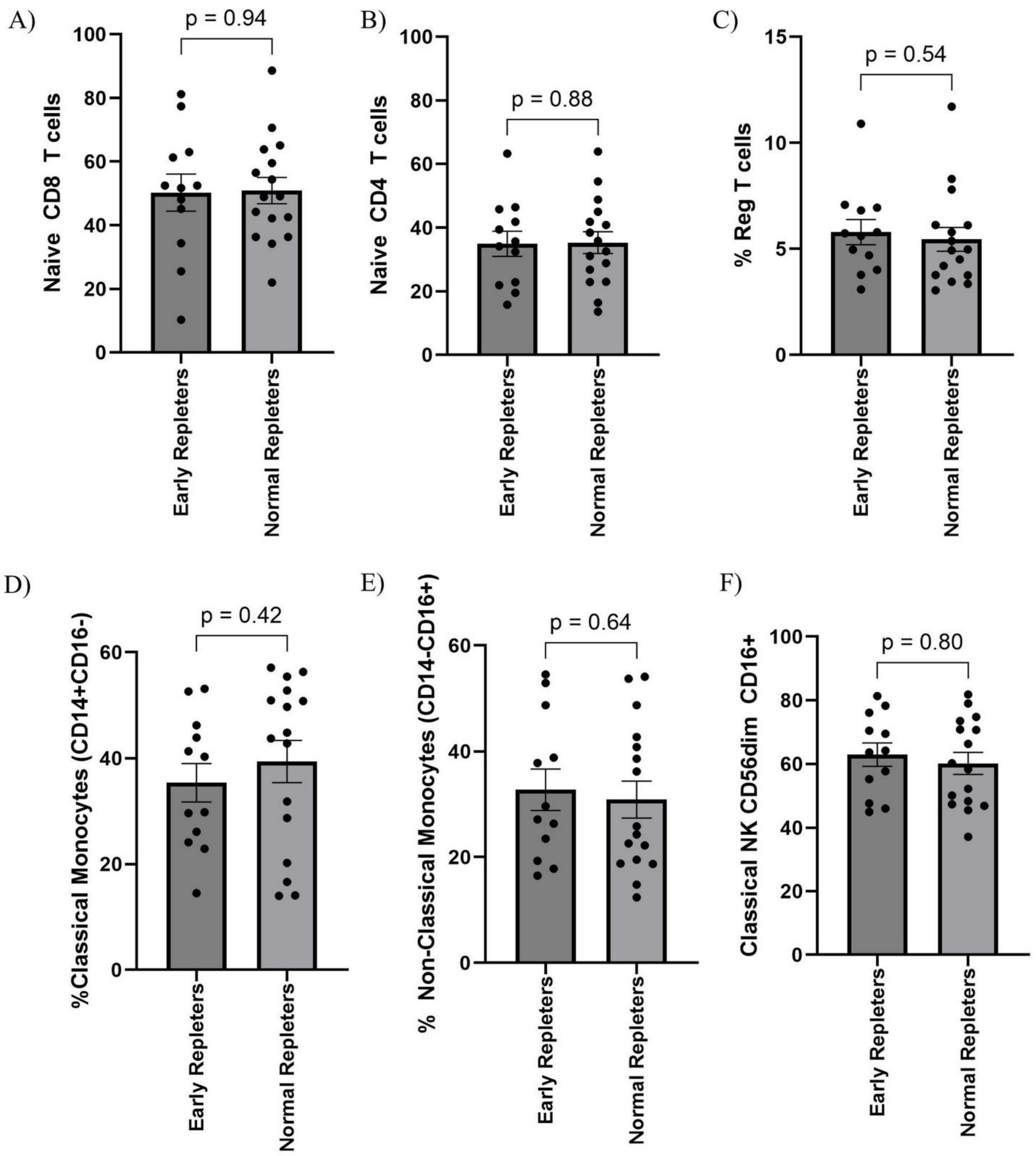

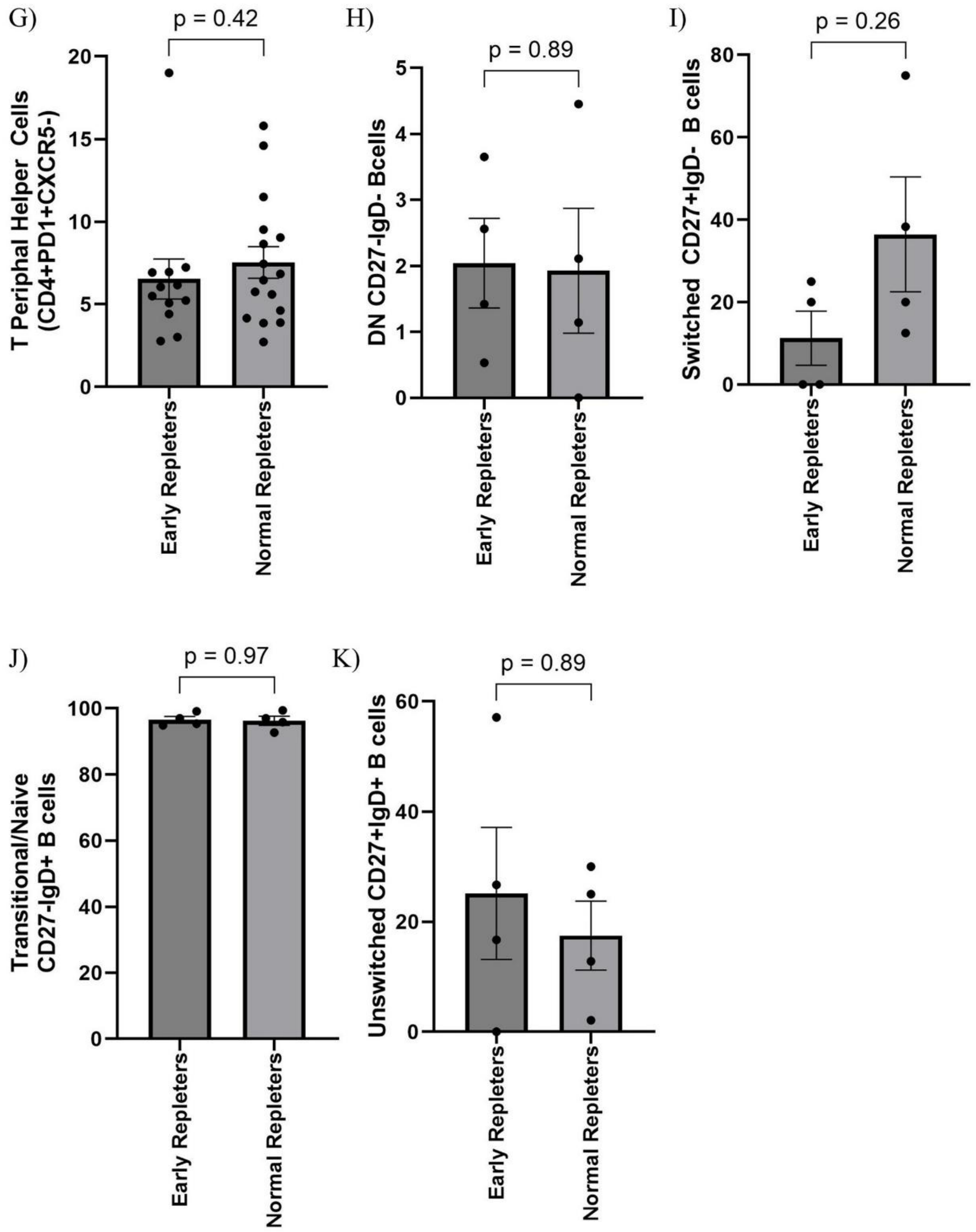

Supplemental Figure 7. The following B cell subsets were assessed: transitional/naïve (CD27$^-$IgD$^+$), switched memory (CD27$^+$IgD$^-$), unswitched memory (CD27$^+$IgD$^+$), and double negative (CD27$^-$IgD$^-$) B cells in multiple sclerosis (MS) patients versus non-responders (NRs). Additionally, no significant differences were observed in the peripheral blood representation of naïve CD4$^+$ T cells, naïve CD8$^+$ T cells, regulatory T cells (CD4$^+$CD25$^+$), or T peripheral helper cells (TPH) (CD4$^+$PD1$^+$CXCR5$^-$). Mean and standard error of the mean (SEM) for each subset are shown. For monocytes, NK cells, and T cells, samples for flow cytometry were tested at 4.7 ± .83 months since infusion for ER and 5.09 ± 2.3 months for NR. For B cells, only flow cytometry samples where a minimum of CD19$^+$ B cells were available for subset analysis were included. For B cell subset analyses, the time since infusion was 5.2 ± .6 months for ER and 7.15 ± 4. months for NR. Markers were not available to differentiate transitional versus naïve B cell subsets further.

Supplemental Figure 8. (A) Mean allele frequency (MAF) distribution of SNPs of interest (2171 SNPs) in normal repleter (n = 23) and early repleter (n = 18) patients, compared to reference MAF of unrelated individuals.

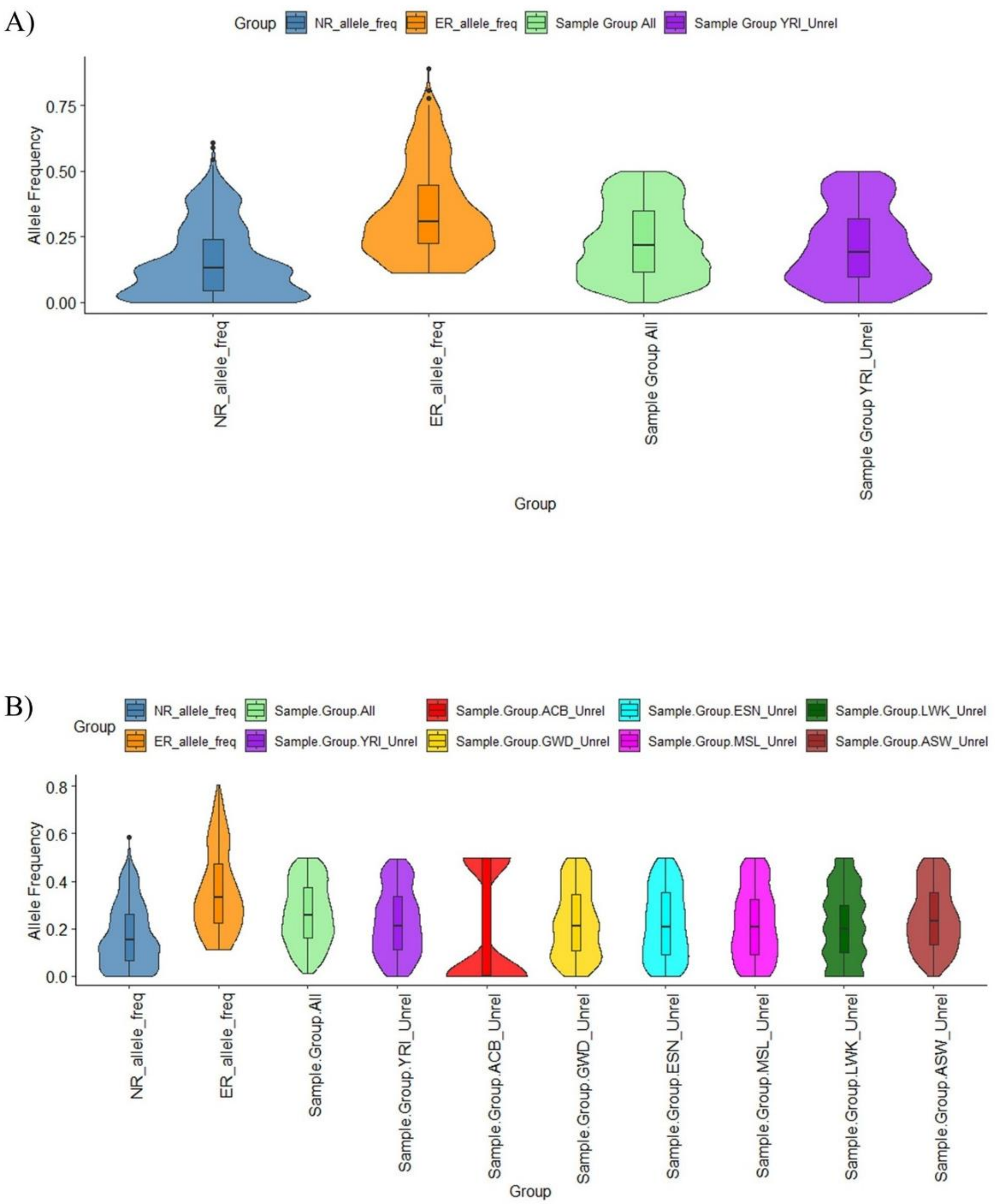

Supplemental Figure 8. The reference populations include the Yoruba people from Ibadan, Nigeria (YRI; n = 58), and a diverse sample group of unrelated individuals (Sample Group All) from the Yoruba in Ibadan, Nigeria (YRI), Han Chinese in Beijing, China (CHB), Japanese in Tokyo, Japan (JPT), and Utah residents with Northern and Western European ancestry (CEU), totaling n = 201. The YRI and all sample reference group MAFs are sourced from the Illumina ImmunoArray Population report. (B) Mean allele frequency (MAF) distribution of SNPs of interest that overlap with reference SNP mean allele frequency data from the Illumina global diversity array (475 SNPs) in normal repleter (n = 23) and early repleter (n = 18) patients compared to Supplemental Figure 6. Mean allele frequency (MAF) distribution of SNPs of interest that overlap with reference SNP mean allele frequency data from the Illumina global diversity array (475 SNPs) in normal repleter (n = 23)

and early repleter (n = 18) patients, compared to reference MAF of unrelated individuals. The reference populations include a diverse sample group of unrelated individuals, totaling n = 2051. The specific reference populations and their sample sizes are as follows: Yoruba people from Ibadan, Nigeria (YRI; n = 85), African Caribbeans in Barbados (ACB; n = 1), Gambian in Western Divisions in the Gambia (GWD; n = 19), Esan in Nigeria (ESN; n = 17), Mende in Sierra Leone (MSL; n = 17), Luhya in Webuye, Kenya (LWK; n = 5), and Americans of African Ancestry in SW USA (ASW; n = 56). The MAFs for these reference populations are sourced from the Illumina global diversity array reference report.
